# The impact of China’s drug regulatory reform on access to orphan drugs: a cross-sectional study of diseases listed in the rare disease catalog

**DOI:** 10.3389/fphar.2026.1833127

**Published:** 2026-06-04

**Authors:** Yipeng Lan, Xiaofeng Lin, Zhe Huang, Wang Zan

**Affiliations:** 1 School of Pharmacy, Chengdu Medical College, Chengdu, China; 2 School of Business Administration, Shenyang Pharmaceutical University, Shenyang, China; 3 Institute of Drug Regulatory Science, Shenyang Pharmaceutical University, Shenyang, China

**Keywords:** accessibility of medicines, China, drug development, drug review and approval, orphan drug, rare disease

## Abstract

**Objective:**

China released two batches of the Rare Disease Catalog in 2018 and 2023, respectively, listing a total of 207 diseases. This study aims to analyze the accessibility of medications for diseases included in the catalog and assess the impact of drug regulatory reforms on the supply of medications for rare diseases.

**Methods:**

This study compiled five categories of information on drugs for 207 rare diseases: basic information on drug availability, the number of drug approvals and production capacity, drug development trends, marketing approval efficiency, and medical insurance coverage. Descriptive statistical analysis was employed.

**Results:**

By the end of 2025, out of 207 rare diseases, 98 (47.3%) had drugs approved in China, with a single company being the exclusive supplier for 37 of these diseases. Between 2015 and 2025, the NMPA approved a total of 2,007 Investigational New Drug (IND) applications and 1,134 New Drug Applications (NDAs) for rare diseases, showing an overall upward trend. Following the publication of the catalog, the median review time for NDA applications for rare disease drugs was reduced by 79 days compared to pre-publication (516 vs. 595, P < 0.001); for drugs eligible for Priority Review and Approval (PRA), the median review time was reduced by 73 days compared to non-PRA drugs (493 vs. 566, P < 0.001). The time difference between the market launches of the same drug in China and the United States has been reduced by 1.52 years (5.53 vs. 4.01, P < 0.001). Of the 263 drugs used to treat 98 rare diseases, 181 were included in the medical insurance coverage, and the time to inclusion in the insurance coverage was reduced by 1.01 years following the publication of the directory (2.09 vs. 1.07, P < 0.001).

**Conclusion:**

With the release of the rare disease catalog and the advancement of drug regulatory reforms, access to orphan drugs in China has improved. However, more than half of all rare diseases still face the dilemma of having no available treatments. It is recommended that the coordination mechanism between drug regulation and medical insurance be maintained to better meet patients’ clinical medication needs.

## 1 Introduction

Rare diseases are a collective term for a group of diseases with a very low prevalence and a small total number of patients. Despite the small number of patients with a single rare disease, as a group, the overall number of people affected is enormous (David and Gareth, 2024). Globally, more than 7,000 rare diseases are currently known, affecting more than 300 million people (Baynam et al., 2024). Due to the worldwide variation in epidemiologic data on rare diseases, the criteria for defining rare diseases vary among the major countries or regions of the world (Supplementary Table S1). Unlike countries or regions such as the United States, Europe, and Japan, so far, China has not officially clarified the criteria for defining the types of rare diseases. However, the National Health Commission (NHC), in conjunction with other departments, released two batches of Rare Disease Catalogs in 2018 and 2023, respectively, which contained a total of 207 diseases (National Health Commission, 2018; National Health Commission, 2018). The release of these two disease catalogs provides a crucial reference for the diagnosis and treatment of rare diseases, drug development, and the formulation of drug policy in China.

“Difficulty in diagnosis” and “difficulty in securing medication” are the two major mountains straddling the road of treatment for patients with rare diseases (Han et al., 2022). One study pointed out that the average number of years from the first visit to the final diagnosis of rare disease patients in China will reach 4.26 years, and even more than 40% of patients have been misdiagnosed at least once (Zhao et al., 2023). Even after overcoming the mountain of “diagnosis”, patients with rare diseases still face difficulties in accessing drugs. In terms of access to drugs, only 5% of rare diseases have a drug treatment program, so rare disease drugs are also figuratively called “orphan drugs” (Wen et al., 2025; Costa et al., 2024). For a long time, Chinese patients have faced multiple challenges in accessing orphan drugs, specifically manifested as: no available treatment after disease diagnosis, drugs already marketed overseas not being available domestically, marketed drugs lacking registration for rare disease indications, and treatment drugs not being included in the medical insurance reimbursement system (Chen et al., 2024; Peng et al., 2024; Wang et al., 2024).

**FIGURE 1:**
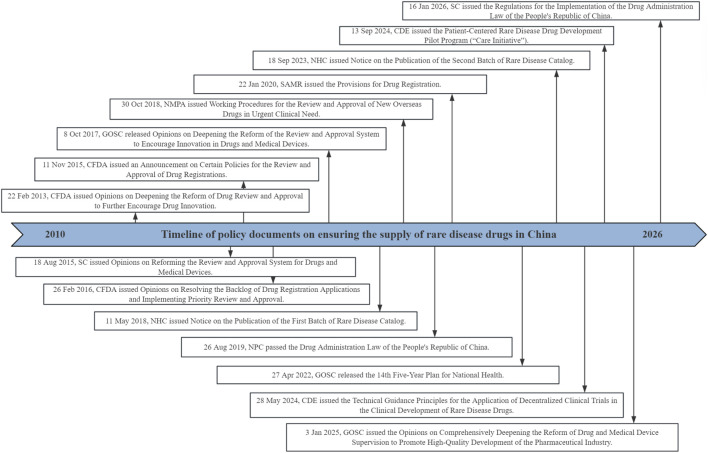
Timeline of policy documents on ensuring the supply of rare disease drugs in China. (CFDA, China Food and Drug Administration (changed to the National Medical Products Administration in 2018); SC, State Council; GOSC, General Office of the State Council; NHC, National Health Commission; NMPA, National Medical Products Administration; NPC, National People’s Congress; SAMR, State Administration of Market Regulation; CDE, Center for Drug Evaluation).

Fortunately, to address these challenges, the Chinese government has placed a high priority on ensuring the supply security of orphan drugs, introducing a series of policies across all stages—from research and development (R&D) to registration, supply, and regulation (Figure 1). Before the drug regulatory reform in 2015, the relevant authorities proposed to expedite the review of registration applications for innovative drugs needed for rare diseases, but no specific guidelines were given (Liu et al., 2024). And after launching the drug regulatory reform in 2015, the Chinese government aimed to improve the accessibility of rare disease drugs through a series of regulatory initiatives, such as the priority review and approval (China Food and Drug Administration, 2016), conditional approval (State Council General Office, 2017), the formulation of rare disease catalogs (2018 and 2023), and decentralized clinical trials (2024) (National Health Commission, 2018; National Health Commission, 2018; China Food and Drug Administration, 2016; State Council General Office, 2017; Center for Drug Evaluation, 2024). More information about China’s rare disease drug assurance policies is shown in Supplementary Table S2.

The accessibility of medicines for rare diseases continues to receive attention from scholars at home and abroad. The majority of published studies have analyzed the R&D, approval, and use of orphan drugs in countries such as the United States and Europe (Bouwman et al., 2024; Monge et al., 2022; Michaeli et al., 2023; Kao et al., 2025). Some Chinese scholars have conducted preliminary research on China’s policies for ensuring the supply security of orphan drugs, as well as the current status of drug accessibility and utilization (Liu et al., 2024; Wang et al., 2022; Chen et al., 2024; Liu et al., 2023). However, existing research primarily consists of qualitative descriptions or cross-sectional analyses at specific points in time. Quantitative assessments of trends in drug accessibility following the release of the two editions of the Rare Disease Catalog in 2018 and 2023—particularly regarding the review and approval dynamics of orphan drugs, post-market access, and medical insurance coverage under the new drug regulatory policies—remain relatively limited.

Given this context, this paper aims to systematically analyze the R&D trends and current accessibility status of drugs corresponding to diseases listed in China’s two editions of the Rare Disease Catalog. It further assesses the actual impact of recent drug regulatory reforms on orphan drug accessibility. The research findings are intended to provide data support and decision-making references for stakeholders, including pharmaceutical R&D companies, rare disease patient groups, drug regulatory authorities, and medical insurance departments.

## 2 Methods

### 2.1 Data sources and extraction

This is a cross-sectional study of orphan drug accessibility in China with a cut-off date of 31 December 2025, for inclusion of data. The World Health Organization (WHO) stated that drug accessibility includes both drug availability and affordability (World Health Organization, 2012). Therefore, this study used the Center for Drug Evaluation (CDE) publicly available data and the Yaozhi Database (a pharmaceutical commercial database widely used in China) to retrieve five aspects of the characteristics of drugs corresponding to 207 rare diseases: the basic situation of drug availability, the number of drug approvals and production supply capacity, drug development trends, drug registration and marketing approval, and medical insurance access (Center for Drug Evaluation, 2025; Yaozhi, 2025).

The basic information on drug availability incorporated the name of the drug, the source of the drug (domestic or imported), the marketing authorization holder (MAH), and the time of first marketing approval. Beyond this basic information, the analysis of the number of drug approvals and production capacity also covers approval numbers, dosage forms, and manufacturer information. Drug development trends mainly incorporate relevant drug investigational new drug (IND) and new drug application (NDA) information, including: IND application type, IND acceptance date, IND approval date, NDA application type, NDA acceptance date, and NDA approval date. Drug registration and marketing approval were incorporated in addition to the key time points mentioned above for drug development to market, and also extracted the accelerated drug marketing and registration procedures (e.g., PRA) for the drugs used. Medical insurance access information included whether or not it was included in medical insurance, the type of medical insurance, when it was first included in medical insurance, and the winning bid prices per unit of the drug before and after it was included in medical insurance. Moreover, the research team examined the current status of drugs approved by the U.S. Food and Drug Administration (FDA) for the same diseases marketed in the United States to attempt a comparison between China and the United States (U.S. Food and Drug Administration, 2025). Furthermore, the research team examined the approval status and characteristics of drugs prior to the publication of the two rare disease catalogs, in an effort to compare drug accessibility before and after the regulatory reforms. The above information helps to provide a comprehensive picture of orphan drug accessibility in China.

**FIGURE 2:**
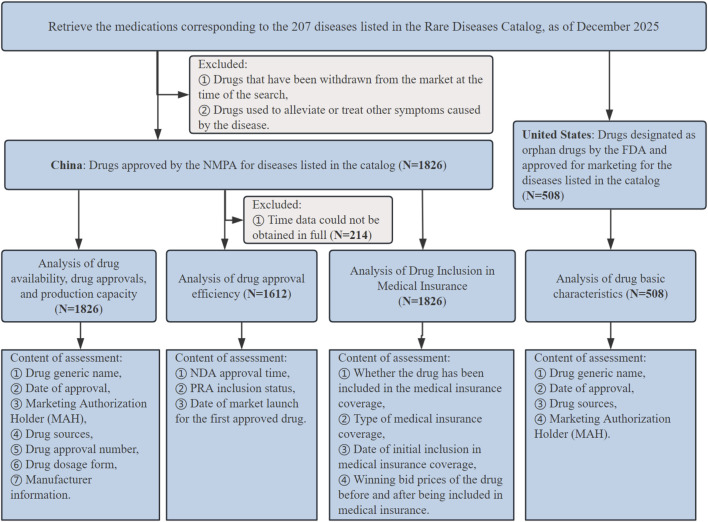
The flowchart of the study. (NMPA, National Medical Products Administration; FDA, U.S. Food and Drug Administration; NDA, new drug application; PRA, priority review and approval).

This study includes drugs that have been approved by the NMPA and are already on the market; drugs for which companies have submitted marketing applications but are still under review are excluded. Drugs for which the filing dates and key information cannot be verified were also excluded. The detailed research flowchart is shown in Figure 2. Data extraction took place from January to February 2026, and was done independently by two researchers and cross-checked. When differences of opinion were encountered, they were discussed and agreed upon by all researchers in a consensus meeting of the research team.

### 2.2 Data analysis

This study used descriptive statistical analysis. Categorical variables were mainly expressed in terms of frequency (percentage), median, and interquartile range (IQR). This study used Fisher’s precision probability test to analyze the distribution of each categorical variable, and the Mann-Whitney U test to analyze the characteristics of orphan drug marketing approval and medical insurance access pre- and post-catalog release. All data required for the study were stored in Microsoft Excel 2022 and analyzed using IBM SPSS Statistics 19; Origin 2019b was used for graphing when necessary. A two-tailed P-value below 0.05 was considered statistically significant in this study.

### 2.3 Patient and public involvement

All material in this study was derived from official public data and open-access information and therefore did not require a research license or ethics committee assessment. Appropriate standards of scientific practice and research ethics were followed throughout the study.

## 3 Results

### 3.1 Basic information on drug availability

**FIGURE 3:**
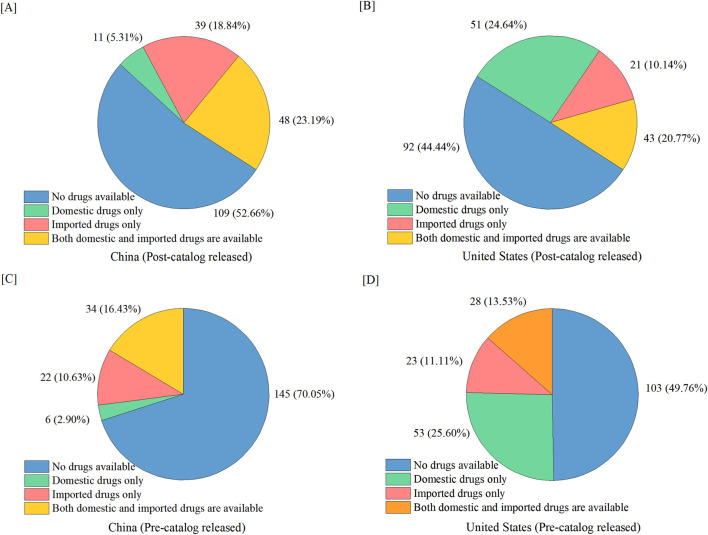
Overview of the availability of rare disease drugs in China and the United States. (**(A)** represents the availability of rare disease drugs in China post-catalog release; **(B)** represents the availability of rare disease drugs in the United States post-catalog release; **(C)** represents the availability of rare disease drugs in China pre-catalog release; **(D)** represents the availability of rare disease drugs in the United States pre-catalog release. Data post-catalog release refers to survey data as of December 31, 2025; data pre-catalog release refers to data as of the date of publication of the two rare disease catalogs. Data sourced from the Yozhi data website).

As of 31 December 2025, 98 of the 207 rare diseases identified by the NHC have available NMPA-approved therapeutic drugs, and the remaining 109 diseases do not yet have available therapeutic drugs (Figure 3A; Supplementary Table S3). Of these 98 diseases with available drugs, 11 diseases were treated with domestically produced drugs, 39 diseases had imported counterparts, and another 48 diseases had both domestically produced and imported drugs. For the United States, for the same 207 diseases, 115 diseases had FDA-approved available drugs, and the remaining 92 diseases had no available therapeutic drugs (Figure 3B). Among the drugs corresponding to these 115 diseases, 51 diseases had U.S. domestically produced drugs, 21 diseases required imported drugs, and 43 diseases had both domestically produced and imported drugs. It was found by Fisher’s precision probability test that there was a difference between China and the United States in terms of the source of drug availability (P < 0.001).

To gain a clear understanding of how the availability of medications for the diseases listed in China’s rare disease catalog has changed since its release, this study conducted a data search and analysis comparing drug availability in China and the United States prior to the catalog’s publication (Figures 3C,D). Compared to the period before catalog publication, by the end of 2025, 36 previously untreatable rare diseases had received approved drugs in China (145 vs. 109). During the same period, the United States approved corresponding drugs for 11 rare diseases (103 vs. 92). For more information on drug availability data for the 207 rare diseases listed in the catalog prior to its release in both China and the United States, see Supplementary Table S4.

Furthermore, the research team analyzed the source of the first-approved drug for each disease for which treatments were available in both China and the United States. Of the 98 rare diseases for which drugs are available in China, only 25 (25.77%) of the drugs first approved for marketing originated from China, while 73 (74.49%) were imported. In contrast, in the United States, of the 115 rare diseases with FDA-approved drugs, 73 (63.48%) of the drugs first approved for marketing originated in the United States, while 42 (36.52%) were imported. After Fisher’s precision probability test, the difference between the two countries in terms of the source of drugs available for the disease was significant (P < 0.001). Therefore, we consider that China was more dependent on imported drugs in terms of access to rare diseases.

### 3.2 Number of drug approvals and production supply capacity

The number of drug approvals and production capacity are important factors in determining drug availability (Lan et al., 2024). Before the publication of the catalog, only 62 diseases had corresponding therapeutic drugs. However, for 10 of these diseases, there was only one approved drug, accounting for 16.13%; for another 22 diseases, the corresponding drugs were exclusively manufactured products, accounting for 35.48% (Table 1). Following the release of the catalog, as of the end of 2025, in terms of the number of drug approvals, of the 98 rare diseases for which drugs were available in China, 63 had fewer than 10 approvals, accounting for 64.28%. Of these, 26 diseases had only one drug approval, as shown in Supplementary Table S3. In terms of the number of pharmaceutical manufacturers, drugs for 75 (76.53%) diseases have fewer than 10 manufacturers, and drugs for 60 (61.22%) diseases have fewer than 5 manufacturers. Among these, 37 diseases have only one supplier of the corresponding drug. See Supplementary Table S3 for more information on these diseases. A comparison of the number of drug approvals and production capacity before and after the catalog release reveals that 36 new drugs have been approved for diseases for which no treatments were previously available. However, the market still faces the reality that single-product and exclusive-production products account for a high proportion of the total.

**TABLE 1:** Number of drug approvals and production capacity for rare disease drugs in China before and after the publication of the catalog.

​	Number before the catalog release No. (%)	Number after the catalog release No. (%)
Number of drug approvals		
≥10	21/62 (33.87%)	35/98 (35.71%)
≥5-<10	11/62 (17.74%)	10/98 (10.20%)
≥2-<5	20/62 (32.26%)	27/98 (27.55%)
Only 1	10/62 (16.13%)	26/98 (26.53%)
Number of drug manufacturers		
≥10	15/62 (24.19%)	23/98 (23.47%)
≥5-<10	10/62 (16.13%)	15/98 (15.31%)
≥2-<5	15/62 (24.19%)	23/98 (23.47%)
Only 1	22/62 (35.48%)	37/98 (37.76%)

“Number of drug approvals” refers to the number of drugs that have received an NMPA approval number, i.e., the number of rare diseases for which at least one drug has been approved; “Number of drug manufacturers” refers to the distribution of rare diseases by the number of pharmaceutical manufacturers. Data sourced from the Yozhi data website.

### 3.3 Drug development trends

Drug R&D is a key indicator of a country’s pharmaceutical industry development and is an important measure of drug availability (Sertkaya et al., 2024). The number of IND and NDA approvals is an important indicator of drug development trends (Lan et al., 2024).

**FIGURE 4:**
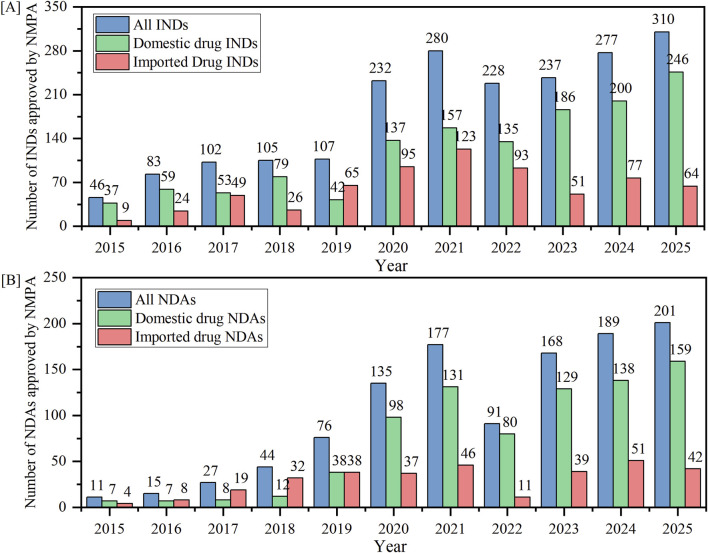
Number of INDs and NDAs for rare disease drugs approved by the NMPA from 2015 to 2025. (**(A)** represents the number of INDs for rare disease drugs approved by the NMPA; **(B)** represents the number of NDAs for rare disease drugs approved by the NMPA. The data represent the number of drugs for which applicants submitted applications and obtained NMPA approval during the year in question, as of the time of the data query for this study. NMPA, national medical products administration; IND, investigational new drug; NDA, new drug application. Data sourced from the Yozhi data website).

From January 2015 to September 2025, the NMPA approved 2007 INDs for rare diseases, including 1,331 domestic drugs and 676 imported drugs. As shown in Figure 4A, the number of INDs for rare disease drugs in China has increased in recent years, especially after the NHC published the First Catalog of Rare Diseases in 2018. Additionally, Supplementary Table S5 reveals that among rare disease drug clinical trials approved by the NMPA in recent years, Class I drug applications accounted for 51.87%. Notably, domestically developed drugs showed a significantly higher proportion of Class I new drug development compared to imported drugs (58.30% vs. 39.20%, P < 0.001).

In terms of drug NDA applications, from January 2015 to December 2025, the NMPA received and approved 1,134 applications for rare disease drugs, including 807 domestic drugs and 327 imported drugs (Figure 4B). Since 2015, the number of orphan drug NDAs approved has increased overall, especially for domestic drugs. However, as shown in Supplementary Table S5, the number of approved Class I orphan drugs is low, at only 25 (2.61%).

### 3.4 Drug registration and marketing approval

**FIGURE 5:**
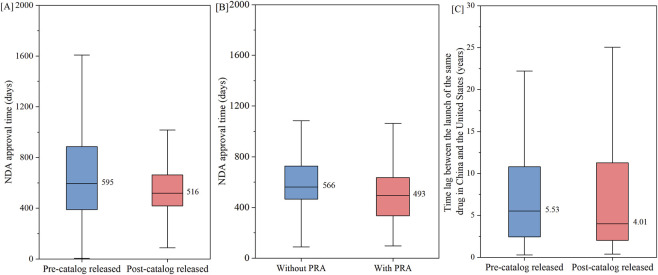
Analysis of time to market approval and lags for rare disease drugs. (**(A)** represents the NDA approval time pre- and post-catalog release; **(B)** represents the NDA approval time with or without PRA post-catalog release; **(C)** represents the time lag between the launch of the same drug in China and the United States pre- and post-catalog release. NDA approval time was defined as the time from the date of submission of the NDA application for a drug to its approval by the NMPA for marketing. NDA, new drug application; PRA, priority review and approval).

The study analyzed the NDA approval time of drugs that have already been approved for marketing. After the rare disease drug catalog was published, the median review time for rare disease drugs decreased by 79 days (516 vs. 595, P < 0.001) (Figure 5A). In 2016, the NMPA began implementing the PRA, which included innovative drugs and modified new drugs for the treatment of diseases such as short-supply drugs, drugs for major infectious diseases, and rare diseases in its review scope (China Food and Drug Administration, 2016). This study found that, after the rare disease drug catalog was released, the median NDA approval time for drugs included in the PRA decreased by 73 days (493 vs. 566, P < 0.001) (Figure 5B).

To further analyze China’s progress in accelerating the approval of orphan drugs, this study compared the time it took for the same drugs to be approved in China and the United States (Figure 5C). The results showed that, before the catalog was released, China’s orphan drug marketing time lagged behind the United States by 5.53 years. After the catalog was released, the time lag was reduced to 4.01 years. This 1.52-year reduction (5.53 vs. 4.01, P < 0.001) precisely verified the achievements of China’s drug regulatory reform in promoting the availability of rare disease drugs.

### 3.5 Medical insurance access

As of the time of data collection, China was implementing the 2025 version of the National Medicare Reimbursement Drug List (NMRDL), which was released on 7 December 2025 (National Healthcare Security Administration, 2025). This study found that of the 263 drugs used to treat 98 rare diseases, 181 were included in this NMRDL, with a medical insurance access rate of 68.82%. Of these, 19 drugs (10.49%) were included in Class A of NMRDL, and 162 drugs (89.50%) were included in Class B of NMRDL. The research team analyzed the length of medical insurance access for orphan drugs before and after the release of the rare disease catalog. After the release of the rare disease catalog, the length of medical insurance access for drugs corresponding to diseases in the catalog was 1.01 years shorter than before the catalog was released (2.09 vs. 1.07, P < 0.001). Additionally, the research team analyzed the rare diseases added to the annual medical insurance directory after the first rare diseases catalog was released (Table 2). The median time for rare disease drugs to be approved for inclusion in the medical insurance program decreased from 2.06 years in 2019 to 1.16 years in 2023 (P < 0.001). Additionally, the median price decrease for these drugs increased from 40.87% in 2019 to 59.77% in 2023 (P < 0.001).

**TABLE 2:** Rare disease drugs newly added to the National Medical insurance reimbursement list from 2019 to 2023.

Year	Increase quantity	Median time of inclusion in medical insurance (IQR) Years	Median unit price decrease (IQR, %)
2019	18	2.06 (1.67–2.77)	40.87 (25.32–57.48)
2020	16	1.02 (0.64–1.52)	40.65 (35.85–55.63)
2021	11	1.18 (0.93–1.34)	53.94 (43.07–68.88)
2022	14	1.79 (1.01–2.80)	52.50 (35.45–59.55)
2023	17	1.16 (0.65–2.65)	59.77 (45.52–74.82)

Seven new drugs were added to the national medical insurance coverage in 2024, and five in 2025. Due to the small sample size, these figures are not suitable for calculating medians or quartiles. The drug price reduction refers to the change in the winning bid price of the drug in Sichuan Province before and after its inclusion in the medical insurance coverage, as retrieved from the Yaozhi data website.

However, this study also found that none of the drugs for the 32 rare diseases were included in the medical insurance program (Supplementary Table S6). Some of these drugs have high unit prices (e.g., Idursulfase Beta Injection, Maralixibat Chloride Oral Solution, and Naxitamab Injection), which increases patients’ economic burden when acquiring these drugs.

## 4 Discussion

This cross-sectional study revealed the current state of the rare disease drug supply in China, as well as the positive outcomes in access to rare disease drugs since China’s drug regulatory reforms.

In terms of drug availability, since the publication of the Rare Disease Catalog, 36 additional diseases have received NMPA-approved medications, resolving the dilemma of “no available treatments”. As of December 2025, 98 diseases have received NMPA-approved treatments; however, for 39 of these diseases, the corresponding medications must be imported entirely, accounting for 39.80%. Although this result decreased compared to Peng et al.'s earlier study, China still relies heavily on imported drugs for rare disease treatment (Peng et al., 2023). After comparing with the United States, it was found that the current situation of rare disease drugs being available abroad but not approved in China still exists. As shown in Figure 1, there were 19 drugs available in the United States that were not available in China. To address the issue of having drugs available in other countries but not in China, the NMPA has developed the “Work Program for the Temporary Importation of Urgently Needed Clinical Drugs” (National Health Commission, 2022). This plan states that for drugs available in other countries but not in China, the NMPA will issue a “consent to import” letter within three business days for drugs that meet the requirements, thereby exempting them from port inspection. For example, Clobazam, used to treat Lennox-Gastaut syndrome, has benefited patients with this condition in China through this program (National Health Commission, 2022).

This study analyzed the production and supply capacity of rare disease drugs, which has been rarely addressed in other studies. In a previous study published by the author, it was noted that the limited number of drug approvals and the relatively small number of pharmaceutical manufacturers were among the potential causes of drug shortages (Lan et al., 2024). According to the data from this study, of the 95 diseases for which drugs were available, 35 had drugs produced exclusively, and 23 had drugs for which there was only one approved application in China. For these medications, which are produced in limited quantities and have an imbalance in supply and demand, especially for products that are produced exclusively, it is necessary to strengthen market regulation and provide more support for R&D (Mao et al., 2022; Kwisda et al., 2025). On the one hand, it is necessary to accelerate the approval of rare disease drugs by leveraging PRA, CA, and other support measures to meet clinical needs. On the other hand, effective monitoring of drug production and supply is essential, and a multi-channel system for drug procurement should be considered when necessary (Lan et al., 2024).

In terms of drug development, orphan drugs face greater R&D challenges, lower economic benefits, and smaller target markets. This results in fewer companies investing in the development of these drugs, and fewer companies are motivated to develop, produce, and register these products (Costa et al., 2025; Ding et al., 2025). Fortunately, this study found that in recent years, the development of drugs for rare diseases has gradually shifted from lagging behind international trends to moving toward global synchronized development. Positive policies are an important incentive for drug research and development. As early as 2018, the CDE published the “Announcement on Adjusting the Drug Clinical Trial Review and Approval Procedure,” which clearly stipulated that drug registration clinical trials would be approved under the licensing model (Center for Drug Evaluation, 2018). The CDE will decide within 60 working days of IND acceptance whether to approve the clinical trial of the drug. Although this system is not unique to orphan drugs, the implementation of the implied approval system provides important support for the advancement of orphan drug clinical trials and global synchronous development. Additionally, drug regulatory authorities have been continuously optimizing review processes and implementing measures such as PRA to enhance review efficiency, thereby providing robust support for the NDA approval for rare disease drugs (National Medical Products Administration, 2020). However, in contrast to the author’s previous findings on the current state of innovative drug development in China, this study found that the number of orphan drugs approved as Class I new drugs was low (2.38%), and they were mainly concentrated in the treatment of tumors (Lan et al., 2024). This reflected the fact that orphan drug development was homogeneous and that there were unmet clinical needs that needed to be addressed.

Regarding the approval of rare disease drugs, the relevant authorities have further accelerated the approval efficiency of rare disease drugs since the release of the first rare disease catalog. Unlike the U.S. Orphan Drug Act’s rare disease designation system, China employs a catalog-based system for rare disease designation. Diseases included in the catalog are officially recognized as rare diseases, and the corresponding drugs may be considered for PRA support. According to the PRA implementation requirements, the review time for drugs included in the PRA is shortened from 200 days to 130 days for standard reviews. For clinical emergency drugs that were approved in other countries but not in China, the review time is shortened to 70 days (China Food and Drug Administration, 2016). The PRA’s support measures, such as priority review and rolling submission of materials, greatly accelerate the approval of orphan drugs. In terms of the international comparison of orphan drug launch times, Liu et al. found that the time lag for the launch of the same drug in China was 5.90 years behind the United States, based on the data of drugs launched from 2017 to 2022 (Liu et al., 2023). However, when we extended the data to 2025 after the release of the rare disease directory, the time lag for the launch of orphan drugs in China was reduced to 4.01 years behind the United States. Therefore, the results of the data comparison in this study and in previously published studies both indicated that the current Chinese drug regulatory authorities have accelerated the approval efficiency of rare disease drugs.

To improve access to drugs, in addition to accelerating the approval process to increase availability, another important task is to lower drug prices to increase affordability (Chen et al., 2024). At the national level, affordability is primarily reflected in drug medical insurance access (Jakovljevic et al., 2023). This study found that after the rare disease catalog was released, the median time for drug access under medical insurance decreased by 1.01 years (2.09 V S. 1.08, P < 0.001). Additionally, many expensive rare disease drugs have been approved through insurance negotiations, allowing more patients to receive effective treatment. For example, the price of the Eculizumab Injection, used to treat general myathenic gravis, decreased by 86.75% (from 19,000 to 2,518 yuan) after being included in the 2023 medical insurance negotiations. However, 46 drugs for 32 diseases have not yet been included in the NMRDL (Supplementary Table S6). Of these, 80.43% (37/46) are imported drugs, while 19.57% (9/46) are domestically produced drugs. Further analysis revealed that 21 of these 37 imported drugs (56.76%) were approved in China through overseas clinical trials or clinical trial exemptions. These drugs faced greater challenges in terms of being included in the medical insurance program due to the lack of complete evidence of their effectiveness in Chinese populations. In China, due to the uncertainty of the NMRDL directory adjustment cycle and the time it takes for drugs to be approved, it takes a long time to conduct clinical trials to obtain effective data and make accurate judgments about the actual efficacy and drug economics of new drugs (Peng et al., 2024). To avoid the inclusion of ineffective rare disease drugs in the medical insurance program, which would waste medical insurance funds or cause the public to lose confidence in government agencies due to poor efficacy and side effects, the medical insurance access process will be more stringent for drugs that have not completed a confirmatory clinical trial.

Overall, following the implementation of the rare disease catalog and corresponding regulatory measures, there have been positive changes in the accessibility of orphan drugs in China. However, considering the current diagnostic, treatment, and medication needs of rare disease patients in China, as well as the practical requirements for pharmaceutical innovation and development, this paper proposes the following recommendations to further enhance access to rare disease drugs.

(i) Update the rare disease catalog quickly and strengthen its role in guiding the development of drugs for treating diseases.

The rare disease catalog plays a positive role in incentivizing orphan drug development. However, the 5-year gap between the release of the first and second catalogs, coupled with the inflexibility of the adjustment mechanism, may hinder the development and market launch of treatments for certain conditions, potentially leading to drug shortages in specific disease areas. It is recommended to further optimize the development of the rare disease catalog and strengthen its guiding role in the treatment of special diseases. On one hand, regarding the criteria for rare disease catalog recognition, the NHC may increase consideration of the availability of marketed drugs in relevant disease areas, prioritizing the inclusion of rare diseases with no or few treatment options to enhance its guiding role in the therapeutic field (Mo et al., 2025; Miller et al., 2024). On the other hand, it is recommended to promote incentives for the R&D of urgently needed rare disease drugs, such as implementing the incentive measures for rare disease drug development outlined in the 2026 version of the Implementing Regulations of the Drug Administration Law (State Council, 2026). This administrative regulation stipulates that government authorities may grant a market exclusivity period of up to 7 years for eligible rare disease therapeutic drugs. This measure will help accelerate the innovative R&D and market launch of rare disease drugs in China.

(ii) Implement the “patient-centered drug development” concept and encourage the development of orphan drugs using relevant incentive policies.

In September 2024, the CDE released the Pilot Program for Patient-Centered Rare Disease Drug Development (“Care Initiative”), aiming to integrate patient experiences related to clinical benefit-risk assessment into the clinical development of rare disease drugs (Center for Drug Evaluation, 2024). As of October 2025, ten drugs for treating rare diseases, including Fitusiran Injection and Eliglustat Tartrate Capsules, have been included in the Care Initiative. During the development of these drugs, the CDE maintained close communication with the applicants, providing timely feedback on issues such as the applicants’ plans for patient involvement in drug development and the methods for applying patient experience data to drug development. This ultimately facilitated the market launch of these rare disease drugs.

(iii) Strengthen post-market evaluation and oversight of rare disease drugs and promptly include eligible drugs in the medical insurance system.

Given the small patient populations for rare diseases and the limited pre-marketing clinical research data, post-marketing evaluation based on real-world data (RWD) becomes particularly crucial (Asano et al., 2025). Additionally, existing medical insurance access criteria require reliable foundational data to support research. However, the actual efficacy and pharmacoeconomic evaluation of newly launched rare disease drugs require extensive clinical trials to obtain efficacy data before accurate assessments can be made, particularly for drugs that have received accelerated approval or been imported into China without undergoing clinical trials (Jakovljevic et al., 2023). Therefore, it is recommended to improve the post-marketing research and risk monitoring system for drugs based on real-world evidence and to strengthen post-marketing evaluation and regulatory oversight of rare disease drugs. Additionally, it is suggested to comprehensively consider factors such as clinical needs and funding affordability to establish a more streamlined access pathway for rare disease drugs, thereby building a multi-tiered rare disease protection system that benefits more patients.

This study has some limitations. First, due to the difficulty of obtaining clinical data on diseases and drugs, we have not yet analyzed the clinical trial design content of approved drugs. Second, this paper primarily examined the accessibility of rare disease drugs from theoretical perspectives, including drug approval and medical insurance coverage. Future research should also investigate drug accessibility from practical angles, including hospital drug procurement practices and pharmacoeconomic evaluations. Third, the article has not yet conducted an in-depth analysis of the differences in drug accessibility between China and the United States due to the distinct regulatory systems governing pharmaceuticals in the two countries; further international research and comparisons will be conducted in the future. Fourth, China’s work to ensure the supply of rare disease drugs is ongoing, while the data analysis in this article only represents a single point in time. The effects of China’s policies to ensure the supply of rare disease drugs will require further analysis in the future.

## 5 Conclusion

The release of two catalogs of rare diseases, along with the advancement of regulatory reforms, including guidance for drug development, review, and approval, has enhanced the accessibility of rare drugs in China. The availability of drugs for diseases listed in the catalog has improved, with a noticeable increase in drug development trends. Moreover, compared to the period before the catalog was released, the approval time for drug market entry has been significantly shortened. As the availability of drugs increases, more orphan drugs that meet the criteria are included in the medical insurance program, making the drugs more affordable for patients. However, there is still an unmet clinical need for medication. With the continued push of policies and regulatory reforms, the future of improving access to rare disease drugs in China is promising.

## Data Availability

The original contributions presented in the study are included in the article/Supplementary Material, further inquiries can be directed to the corresponding author.

## References

[B1] Asano J. Sugano H. Murakami H. Noguchi A. Ando Y. Uyama Y. (2025). PMDA perspective on use of real-world data and real-world evidence as an external control: recent examples and considerations. Clin. Pharmacol. Ther. 117 (4), 910–919. 10.1002/cpt.3540 39749966 PMC11924144

[B2] Baynam G. Hartman A. L. Letinturier M. C. Bolz-Johnson M. Carrion P. Grady A. C. (2024). Global health for rare diseases through primary care. Lancet Glob. Health 12 (7), e1192–e1199. 10.1016/S2214-109X(24)00134-7 38876765 PMC13271179

[B3] Bouwman L. Sepodes B. Leufkens H. Torre C. (2024). Trends in orphan medicinal products approvals in the european union between 2010-2022. Orphanet J. Rare. Dis. 19 (1), 91. 10.1186/s13023-024-03095-z 38413985 PMC10900541

[B4] Center for Drug Evaluation (2018). Announcement on adjusting the review and approval procedures for drug clinical trials. Available online at: https://www.cde.org.cn/main/policy/view/10d42c2d13ba72df587642f8fd257600 (Accessed March 12, 2026).

[B5] Center for Drug Evaluation (2024). Pilot program for patient-centered rare disease drug development. Available online at: https://www.cde.org.cn/main/news/viewInfoCommon/244dc3661a418359aa12d7cba9bacf5d (Accessed March 12, 2026).

[B6] Center for Drug Evaluation (2024). Technical Guidelines for the Application of Decentralized Clinical Trials in the Clinical Development of Drugs for Rare Diseases.

[B7] Center for Drug Evaluation (2025). Drug receiving varieties information query system. Available online at: https://www.cde.org.cn/main/xxgk/listpage/9f9c74c73e0f8f56a8bfbc646055026d (Accessed March 12, 2026).

[B8] Chen H. D. Xiang Y. L. Tang X. Hu M. (2024). Establishment of a value assessment framework for orphan medicinal products in China. Orphanet J. Rare Dis. 19 (1), 390. 10.1186/s13023-024-03393-6 39428462 PMC11492536

[B9] Chen R. Liu S. Han J. S. Zhou S. H. Liu Y. Chen X. Y. (2024). Trends in rare disease drug development. Nat. Rev. Drug Discov. 23 (3), 168–169. 10.1038/d41573-023-00177-8 37932437

[B10] Chen Y. Chen X. Y. Deng Y. Ding J. X. (2024). Analysis of affordability differences for rare diseases in China: a comparison across disease types and regions. Int. J. Equity Health 23 (1), 64. 10.1186/s12939-024-02137-z 38504266 PMC10953120

[B11] China Food and Drug Administration (2016). Opinions on solving the backlog of drug registration applications and implementing priority review and approval. Available online at: https://www.nmpa.gov.cn/xxgk/fgwj/gzwj/gzwjyp/20160226085101295.html (Accessed March 12, 2026).

[B12] Costa E. Moja L. Wirtz V. J. van den Ham H. A. Huttner B. Magrini N. (2024). Uptake of orphan drugs in the WHO essential medicines lists. B. World Health Organ 102 (1), 22–31. 10.2471/BLT.23.289731 38164340 PMC10753278

[B13] Costa E. Ajith V. Al Khaldi A. F. Isgrò A. Lee K. J. Luigetti R. (2025). Addressing global regulatory challenges in rare disease drug development. Drug Discov. Today 30 (10), 104462. 10.1016/j.drudis.2025.104462 40889637

[B14] David A. Gareth B. (2024). The landscape for rare diseases in 2024. Lancet Glob. Health 12 (3), e341. 10.1016/S2214-109X(24)00056-1 38365397

[B15] Ding J. T. Huang H. Y. Wang Y. N. Hou Y. R. Zhao G. Cai Y. T. (2025). Expediting the integration of China into the global rare disease drug development and approval. Sci. Bull. 70 (13), 2066–2069. 10.1016/j.scib.2025.04.036 40328602

[B16] Han Q. Q. Fu H. T. Chu X. Y. Wen R. X. Zhang M. You T. (2022). Research advances in treatment methods and drug development for rare diseases. Front. Pharmacol. 13, 971541. 10.3389/fphar.2022.971541 36313320 PMC9597619

[B17] Jakovljevic M. Chang H. Y. Pan J. Guo C. Hui J. Hu H. (2023). Successes and challenges of China's health care reform: a four-decade perspective spanning 1985-2023. Cost. Eff. Resour. A 21 (1), 59. 10.1186/s12962-023-00461-9 37649062 PMC10469830

[B18] Kao J. Van de Wiele V. L. Beall R. F. Sarpatwari A. (2025). Impact of risk evaluation and mitigation strategies on generic approvals of US pharmaceutical products. Health Aff. 44 (10), 1285–1290. 10.1377/hlthaff.2024.01476 41052395

[B19] Kwisda S. Kremer M. Sievertsen N. Gassmann O. Hartl D. Schuhmacher A. (2025). Does pharma R&D need a strategic reset? Adapting to a changing US landscape. Drug Discov. Today 30 (9), 104442. 10.1016/j.drudis.2025.104442 40738281

[B20] Lan Y. P. Lin X. F. Chen Q. N. Wang L. Sun L. H. Huang Z. (2024). Drug supply and assurance: a cross-sectional study of drug shortage monitoring varieties in China. BMC Public Health 24 (1), 2048. 10.1186/s12889-024-19361-5 39080661 PMC11289944

[B21] Lan Y. P. Lin X. F. Rao Y. M. Huang Z. (2024). Improving access to domestic innovative medicines: characteristics and trends of approved drugs in China 2010-2024. Drug Discov. Today 29 (12), 104240. 10.1016/j.drudis.2024.104240 39542205

[B22] Liu J. Yu Y. Zhong M. K. Ma C. L. Shao R. (2023). Long way to go: progress of orphan drug accessibility in China from 2017 to 2022. Front. Pharmacol. 14, 1138996. 10.3389/fphar.2023.1138996 36969835 PMC10031016

[B23] Liu M. L. Lu Y. Q. Li J. F. Zhang Y. T. Zhang S. S. Liu Y. S. (2024). Orphan drug policy analysis in China. Front. Pharmacol. 15, 1278710. 10.3389/fphar.2024.1278710 38939834 PMC11208459

[B24] Mao W. H. Jiang H. L. Mossialos E. Chen W. (2022). Improving access to medicines: lessons from 10 years of drug reforms in China, 2009-2020. BMJ Glob. Health 7 (11), e009916. 10.1136/bmjgh-2022-009916 36332928 PMC9639057

[B25] Michaeli T. Jürges H. Michaeli D. T. (2023). FDA approval, clinical trial evidence, efficacy, epidemiology, and price for non-orphan and ultra-rare, rare, and common orphan cancer drug indications: cross sectional analysis. BMJ-Brit. Med. J. 381, e073242. 10.1136/bmj-2022-073242 37160306 PMC10167557

[B26] Miller K. L. Lanthier M. (2024). Orphan drug label expansions: analysis of subsequent rare and common indication approvals. Health Aff. 43 (1), 18–26. 10.1377/hlthaff.2023.00219 38190603

[B27] Mo Q. H. He J. Q. Guo Q. X. Yang Y. (2025). Drug lag and associated factors of orphan drugs approved by the US in China. Front. Pharmacol. 16, 1595497. 10.3389/fphar.2025.1595497 40949125 PMC12425770

[B28] Monge A. N. Sigelman D. W. Temple R. J. Chahal H. S. (2022). Use of US food and drug administration expedited drug development and review programs by orphan and nonorphan novel drugs approved from 2008 to 2021. JAMA Netw. Open 5 (11), e2239336. 10.1001/jamanetworkopen.2022.39336 36318210 PMC9627417

[B29] National Health Commission (2018). Notice of publication of the first catalog of rare diseases. Available online at: https://www.gov.cn/zhengce/zhengceku/2018-12/31/content_5435167.htm (Accessed March 12, 2026).

[B30] National Health Commission (2022). Work program for the temporary importation of urgently needed clinical drugs. Available online at: https://www.gov.cn/zhengce/zhengceku/2022-06/30/content_5698580.htm (Accessed March 12, 2026).

[B31] National Health Commission (2022). Work program on temporary imports of clobazam. Available online at: https://www.nhc.gov.cn/yaozs/c100098/202206/d1c76f741b254636891324dbe2bea891.shtml (Accessed March 12, 2026).

[B32] National Health Commission (2023). Notice of publication of the second catalog of rare diseases. Available online at: https://www.gov.cn/zhengce/zhengceku/202309/content_6905273.htm (Accessed March 12, 2026).

[B33] National Healthcare Security Administration (2025). National drug list for basic medical insurance, work injury insurance and maternity insurance. Available online at: https://www.nhsa.gov.cn/art/2025/12/7/art_104_18970.html (Accessed March 12, 2026).

[B34] National Medical Products Administration (2020). Announcement on the release of three documents, including the work procedures for the review of breakthrough therapeutic drugs (for trial implementation). Available online at: https://www.nmpa.gov.cn/xxgk/fgwj/xzhgfxwj/20200708151701834.html (Accessed March 12, 2026).

[B35] Peng F. F. Zheng H. (2023). Analysis on the marketing trend and approval lag of imported orphan drugs from 2010 to 2021 in China. Ther. Innov. Regul. Sci. 57 (6), 1314–1321. 10.1007/s43441-023-00572-8 37651044

[B36] Peng N. Du C. Y. Gong Y. R. Long X. Wang C. Y. Liu P. C. (2024). Systematic review of the impact of the national medication price negotiated policy on the accessibility of drugs in China, 2016-2024. BMJ Open 14 (12), e087190. 10.1136/bmjopen-2024-087190 39725414 PMC11683965

[B37] Sertkaya A. Beleche T. Jessup A. Sommers B. D. (2024). Costs of drug development and research and development intensity in the US, 2000-2018. JAMA Netw. Open 7 (6), e2415445. 10.1001/jamanetworkopen.2024.15445 38941099 PMC11214120

[B38] State Council (2026). Regulations for the implementation of the drug administration law of the People'S Republic of China. Available online at: https://www.gov.cn/zhengce/zhengceku/202601/content_7056256.htm (Accessed November 16, 2025).

[B39] State Council General Office (2017). Opinions on deepening the reform of the review and approval system to encourage innovation in drugs and medical devices. Available online at: https://www.gov.cn/zhengce/2017-10/08/content_5230105.htm (Accessed March 12, 2026).

[B40] U.S. Food and Drug Administration (2025). Orphan drug designation and approval search system. Available online at: https://www.accessdata.fda.gov/scripts/opdlisting/oopd/index.cfm (Accessed March 12, 2026).

[B41] Wang S. H. Yang Q. Y. Deng L. Lei Q. Yang Y. Q. Ma P. W. (2022). An overview of cancer drugs approved through expedited approval programs and orphan medicine designation globally between 2011 and 2020. Drug Discov. Today 27 (5), 1236–1250. 10.1016/j.drudis.2021.12.021 34971818

[B42] Wang Y. Q. Zhou N. Li B. L. Lv Z. X. Duan S. N. Li X. (2024). Utilization and affordability of health insurance coverage for rare disease drugs in a first-tier city in northeast China from 2018 to 2021: a study based on the health insurance claims database. Int. J. Equity Health 23 (1), 151. 10.1186/s12939-024-02225-0 39085851 PMC11290155

[B43] Wen X. Y. Jin G. Z. Wu C. X. (2025). Visual research of global orphan drug from a bibliometric perspective. Drug Des. Dev. Ther. 19, 4201–4220. 10.2147/DDDT.S506112 PMC12103199 40416799

[B44] World Health Organization (2012). Measuring medicine prices, availability, affordability and price components. Available online at: https://www.who.int/publications/i/item/WHO-PSM-PAR-2008.3 (Accessed March 12, 2026).

[B45] Yaozhi (2025). Yaozhi Database. Available online at: https://www.yaozh.com (Accessed March 12, 2026).

[B46] Zhao Z. Y. Pei Z. Y. Hu A. X. Zhang Y. H. Chen J. (2023). Analysis of incentive policies and initiatives on orphan drug development in China: challenges, reforms and implications. Orphanet J. Rare Dis. 18 (1), 220. 10.1186/s13023-023-02684-8 37501126 PMC10375655

