# Supplementary material for: The impact of China’s drug regulatory reform on access to orphan drugs: a cross-sectional study of diseases listed in the rare disease catalog
Source: Front Pharmacol. 2026 Jun 4;17:1833127. doi: 10.3389/fphar.2026.1833127 (PMC13275702; doi:10.3389/fphar.2026.1833127)
Supplement: Supplementary file 1 [file DataSheet1.pdf]

SUPPLEMENTARY MATERIAL

Title: The Impact of China's Drug Regulatory Reform on Access to Orphan Drugs: A Cross-Sectional Study of Diseases Listed in the Rare Disease Catalog

Authors: Yipeng Lan[1], Xiaofeng Lin[2], Zhe Huang[2,3], Wang Zan[1*]
1 School of Pharmacy, Chengdu Medical College, Chengdu, China
2 School of Business Administration, Shenyang Pharmaceutical University, Shenyang, China
3 Institute of Drug Regulatory Science, Shenyang Pharmaceutical University, Shenyang, China
* Correspondence: Wang Zan (33058766@qq.com)

Table S1. Definition of rare diseases in major countries and regions worldwide

Country or Region	Source of the definition	Definition of Rare Diseases
United States	The Orphan Drug Act	Under the Orphan Drug Act, enacted in 1983, a rare disease is defined as one with a prevalence of less than 7.5 per 100,000 individuals, or one affecting fewer than 200,000 people, or one affecting more than 200,000 people but where the anticipated sales of the treatment are unlikely to recoup the costs of research and development.
European Union	European Union Orphan Drug Regulation	According to the Orphan Medicinal Product Regulation promulgated in 2000, a rare disease refers to a life-threatening or debilitating condition with a prevalence rate below 5 per 100,000 people. It also refers to diseases for which there are currently no marketed drugs available for diagnosis, prevention, or treatment, or diseases for which drugs offer significant advantages over existing products.
Japan	Pharmaceutical Affairs Law	According to the Pharmaceutical Affairs Act revised in 1993, a rare disease refers to a condition affecting fewer than 50,000 individuals or with a prevalence rate below 1 in 2,500; it also includes life-threatening diseases for which no relevant drugs exist for diagnosis, prevention, or treatment, or for which existing products offer no significant advantages.
China	Rare Disease Catalogs	China has yet to officially define rare diseases. The National Health Commission (NHC), in conjunction with other departments, released two batches of Rare Disease Catalogs in 2018 and 2023, respectively, which contained a total of 207 diseases.

Table S2. Important policies related to rare disease drug access issued by the Chinese government in recent years

No.	Policy title	Issuing department	Release time	Key initiatives
1	Opinions on Deepening the Reform of Drug Review and Approval to Further Encourage Drug Innovation	CFDA	February 22, 2013	Accelerated review shall be granted for innovative drug registration applications that demonstrate superior therapeutic effects for major diseases, rare diseases, geriatric conditions, and pediatric diseases; possess independent intellectual property rights; and are included in major national science and technology programs.
2	Opinions on the Reform of the Review and Approval System for Drugs and Medical Devices	SC	August 18, 2015	Accelerate the review and approval of innovative drugs for the prevention and treatment of diseases such as AIDS, malignant tumors, major infectious diseases, and rare diseases.
3	Announcement on Certain Policies for the Review and Approval of Drug Registration	CFDA	November 11, 2015	Innovative drug registration applications for the prevention and treatment of diseases such as AIDS, malignant tumors, major infectious diseases, and rare diseases shall be processed through a separate queue to expedite review and approval.
4	Opinions on Solving the Backlog of Drug Registration Applications and Implementing Priority Review and Approval	CFDA	February 26, 2016	For rare diseases or other special conditions, applications may be submitted during clinical trial registration to reduce the number of clinical trial cases or request an exemption from conducting clinical trials.
5	Opinions on Deepening the Reform of the Review and Approval System to Encourage Drug and Medical Device Innovation	GOSC	October 8, 2017	Relevant national authorities shall publish catalogs of rare diseases and establish a registration system for rare disease patients. Applicants for registration of rare disease treatments may request exemptions from clinical trials. Rare disease treatments approved for marketing

				overseas may be conditionally approved for domestic market entry, provided that companies develop risk management plans and conduct required studies.
6	Notice on the Publication of the First Batch of Rare Disease Catalog	NHC	May 11, 2018	To strengthen the management of rare diseases in China, improve the level of diagnosis and treatment of rare diseases, and safeguard the health rights and interests of patients with rare diseases, the first catalog of rare diseases has been formulated, incorporating 121 diseases.
7	Circular on the Issuance of Technical Guidelines for the Acceptance of Clinical Trial Data from Overseas Pharmaceuticals	NMPA	July 6, 2018	For orphan drug applications, if the assessment determines that the foreign clinical trial data is "partially accepted," the data may be accepted with conditions. Further efficacy and safety data will be collected after the drug is approved for marketing.
8	Working Procedures for the Review and Approval of New Overseas Drugs in Urgent Clinical Need	NMPA	October 30, 2018	A dedicated drug review and approval channel was set up for rare disease drugs that have been launched in the United States, the European Union, or Japan but not in China.
9	Drug Administration Law of the People's Republic of China	NPC	August 26, 2019	New drugs for treating rare and serious diseases, as well as those that are in short supply, should be reviewed and approved first.
10	Provisions for Drug Registration	SAMR	January 22, 2020	Priority review and approval will be given to drugs in short supply that are urgently needed for clinical purposes, as well as to innovative and improved new drugs for the prevention and treatment of major infectious diseases, rare diseases, and other conditions. The time limit for reviewing clinically urgently needed drugs for rare diseases that have been marketed abroad but not in China is 70 days.

11	The 14th Five-Year Plan for National Health	GOSC	April 27, 2022	Deepen the reform of the review and approval system for drugs and medical devices, and accelerate the review and approval of innovative medicines that meet the requirements, drugs in shortage and medical devices that are urgently needed for clinical purposes, and drugs for the treatment of rare diseases.
12	Notice on the Publication of the Second Batch of Rare Disease Catalog	NHC	September 18, 2023	To further enhance the diagnosis and treatment of rare diseases in China and safeguard the health rights of patients with rare diseases, the second batch of the Rare Disease Catalog has been formulated and released, covering 86 diseases.
13	Technical Guidance Principles for the Application of Decentralized Clinical Trials in the Clinical Development of Rare Disease Drugs	CDE	May 28, 2024	Taking into account the characteristics of rare diseases, the guideline provides recommendations on how to apply decentralized clinical trials (DCTs) during the clinical development of rare disease drugs, offering guidance for the scientific and standardized implementation of DCTs in rare disease drug research and development.
14	Patient-Centered Rare Disease Drug Development Pilot Program ("Care Initiative")	CDE	September 13, 2024	Guiding and helping research units to fully consider patient voices throughout the process of developing rare disease drugs. Promoting patient-centered rare disease drug development and listing.
15	Opinions on Comprehensively Deepening the Reform of Drug and Medical Device Supervision to Promote High-Quality Development of the Pharmaceutical Industry	GOSC	January 3, 2025	Accelerate the review and approval of rare disease drugs and medical devices, and exempt eligible innovative rare disease drugs and medical devices from clinical trials. Reduce the number of registration inspection batches for rare disease drugs from three to one, and decrease the sample size per batch from three times to twice the full-item testing requirement. Coordinate import

				registration inspections and post-market surveillance of rare disease drugs based on product risk assessment, and shorten the waiting period for overseas inspections.
16	Guidance Principles for Pharmaceutical Research on Chemical Drugs for Rare Diseases (Draft for Comment)	CDE	August 14, 2025	Enhance the efficiency of rare disease drug R&D, and encourage and accelerate the development and market launch of rare disease drugs.
17	Regulations for the Implementation of the Drug Administration Law of the People's Republic of China	SC	January 16, 2026	The Implementing Regulations stipulate that the state supports the research, development, and innovation of pediatric drugs and drugs for the treatment of rare diseases. For eligible drugs used to treat rare diseases, market exclusivity of up to seven years will be granted if the marketing authorization holder commits to ensuring the supply of the drug.

Note: The publishing department here mainly refers to the department at the top of the list. CFDA, China Food and Drug Administration (changed to the National Medical Products Administration in 2018); SC, State Council; GOSC, General Office of the State Council; NHC, National Health Commission; NMPA, National Medical Products Administration; NPC, National People's Congress; SAMR, State Administration of Market Regulation; CDE, Center for Drug Evaluation.

Table S3. Analysis of the availability of rare disease drugs in China and the United States after the catalog release

No.	Batches	Disease name	China					United States				
			Drugs availabl e	Source of drugs	Number of drug approvals (all)	Number of domestic drug approvals	Number of imported drug approvals	Drugs available	Source of Drugs	Number of drug approvals (all)	Number of domestic drug approvals	Number of imported drug approvals
1	First batch	21-Hydroxyulase deficiency	No	—	0	0	0	No	—	0	0	0
2	First batch	Albinism	No	—	0	0	0	No	—	0	0	0

frontiers

3	First batch	Alport syndrome	No	—	0	0	0	No	—	0	0	0
4	First batch	Amyotrophic lateral sclerosis	Yes	Domestic and imported	50	46	4	Yes	Domestic and imported	8	4	4
5	First batch	Angelman syndrome	No	—	0	0	0	No	—	0	0	0
6	First batch	Arginase deficiency	Yes	Imported	1	0	1	No	—	0	0	0
7	First batch	Asphyxiating thoracic dystrophy（Jeune syndrome）	No	—	0	0	0	No	—	0	0	0
8	First batch	Atypical hemolytic uremic syndrome	Yes	Imported	1	0	1	Yes	Domestic	2	2	0
9	First batch	Autoimmune encephalitis	No	—	0	0	0	No	—	0	0	0
10	First batch	Autoimmune hypophysitis	No	—	0	0	0	No	—	0	0	0
11	First batch	Autoimmune insulin receptopathy（type B insulin resistance）	No	—	0	0	0	No	—	0	0	0
12	First batch	Beta-ketothiolase deficiency	No	—	0	0	0	No	—	0	0	0
13	First batch	Biotinidase deficiency	No	—	0	0	0	No	—	0	0	0
14	First batch	Cardic ion channelopathies	No	—	0	0	0	No	—	0	0	0
15	First batch	Carnitine deficiency	Yes	Domestic and imported	59	58	1	Yes	Domestic	3	3	0
16	First batch	Castleman disease	Yes	Imported	2	0	2	Yes	Domestic	1	1	0
17	First batch	Charcot-marie-tooth	No	—	0	0	0	No	—	0	0	0

		disease										
18	First batch	Citrullinemia	Yes	Imported	1	0	1	No	—	0	0	0
19	First batch	Congenital adrenal hypoplasia	No	—	0	0	0	No	—	0	0	0
20	First batch	Congenital hyperinsulinemic hypoglycemia	No	—	0	0	0	No	—	0	0	0
21	First batch	Congenital myasthenic syndrome	No	—	0	0	0	No	—	0	0	0
22	First batch	Congenital myotonia syndrome （Non-dystrophic myotonia, NDM）	No	—	0	0	0	No	—	0	0	0
23	First batch	Congenital scoliosis	No	—	0	0	0	No	—	0	0	0
24	First batch	Coronary artery ectasia	No	—	0	0	0	No	—	0	0	0
25	First batch	Diamond-blackfan anemia	No	—	0	0	0	No	—	0	0	0
26	First batch	Erdheim-chester disease	No	—	0	0	0	Yes	Domestic	1	1	0
27	First batch	Fabry disease	Yes	Imported	3	0	3	Yes	Domestic	3	3	0
28	First batch	Familial mediterranean fever	Yes	Imported	1	0	1	Yes	Domestic	2	2	0
29	First batch	Fanconi anemia	No	—	0	0	0	No	—	0	0	0
30	First batch	Galactosemia	No	—	0	0	0	No	—	0	0	0
31	First batch	Gaucher’s disease	Yes	Domestic and imported	4	2	2	Yes	Domestic	6	6	0
32	First batch	General myathenic gravis	Yes	Domestic and	5	1	4	Yes	Domestic and	7	5	2

				imported					imported			
33	First batch	Gitelman syndrome	No	—	0	0	0	No	—	0	0	0
34	First batch	Glutaric acidemia type I	No	—	0	0	0	No	—	0	0	0
35	First batch	Glycogen storage disease（type I、II）	Yes	Imported	2	0	2	Yes	Domestic	3	3	0
36	First batch	Hemophilia	Yes	Domestic and imported	117	72	45	Yes	Domestic	28	28	0
37	First batch	Hepatolenticular degeneration（wilson disease）	Yes	Domestic and imported	12	8	4	Yes	Domestic and imported	3	2	1
38	First batch	Hereditary angioedema（HAE）	Yes	Domestic and imported	13	10	3	Yes	Domestic and imported	13	12	1
39	First batch	Hereditary epidermolysis bullosa	No	—	0	0	0	Yes	Domestic and imported	2	1	1
40	First batch	Hereditary fructose intolerance	No	—	0	0	0	No	—	0	0	0
41	First batch	Hereditary hypomagnesemia	Yes	Domestic	6	6	0	No	—	0	0	0
42	First batch	Hereditary multi-infarct dementia（Cerebral autosomal dominant arteriopathy with subcortical infarcts and leukoencephalopathy, CADASIL）	No	—	0	0	0	No	—	0	0	0

43	First batch	Hereditary spastic paraplegia	No	—	0	0	0	No	—	0	0	0
44	First batch	Holocarboxylase synthetase deficiency	No	—	0	0	0	No	—	0	0	0
45	First batch	Homocysteinemia	No	—	0	0	0	No	—	0	0	0
46	First batch	Homozygous hypercholesterolemia	Yes	Domestic and imported	307	281	26	Yes	Domestic and imported	8	7	1
47	First batch	Huntington disease	Yes	Imported	8	0	8	Yes	Domestic and imported	4	3	1
48	First batch	Hyperornithinaemia-hyperammonaemia-hhomocitrullinuria syndrome	Yes	Imported	1	0	1	No	—	0	0	0
49	First batch	Hyperphenylalaninemia	Yes	Domestic	1	1	0	Yes	Domestic	3	3	0
50	First batch	Hypophosphatasia	No	—	0	0	0	Yes	Domestic	1	1	0
51	First batch	Hypophosphatemic rickets	Yes	Domestic and imported	24	17	7	Yes	Imported	2	0	2
52	First batch	Idiopathic Cardiomyopathy	Yes	Imported	1	0	1	Yes	Domestic	5	5	0
53	First batch	Idiopathic hypogonadotropic hypogonadism	Yes	Domestic and imported	53	52	1	Yes	Imported	2	0	2
54	First batch	Idiopathic pulmonary arterial hypertension	Yes	Domestic and imported	54	26	28	Yes	Domestic and imported	18	12	6
55	First batch	Idiopathic pulmonary	Yes	Domestic	22	18	4	Yes	Domestic	3	1	2

		fibrosis		and imported					and imported			
56	First batch	IgG4 related disease	Yes	Imported	1	0	1	No	—	0	0	0
57	First batch	Inborn errors of bile acid synthesis	No	—	0	0	0	Yes	Domestic	1	1	0
58	First batch	Isovaleric acidemia	Yes	Domestic and imported	2	1	1	No	—	0	0	0
59	First batch	Kallmann syndrome	No	—	0	0	0	No	—	0	0	0
60	First batch	Langerhans cell histiocytosis	No	—	0	0	0	No	—	0	0	0
61	First batch	Laron syndrome	No	—	0	0	0	No	—	0	0	0
62	First batch	Leber hereditary optic neuropathy	No	—	0	0	0	No	—	0	0	0
63	First batch	Long chain 3-hydroxyacyl-CoA dehydrogenase deficiency	No	—	0	0	0	No	—	0	0	0
64	First batch	Lymphangioleiomyoma tosis (LAM)	No	—	0	0	0	Yes	Domestic and imported	4	1	3
65	First batch	Lysine urinary protein intolerance	No	—	0	0	0	No	—	0	0	0
66	First batch	Lysosomal acid lipase deficiency	No	—	0	0	0	Yes	Domestic	1	1	0
67	First batch	Maple syrup urine disease	No	—	0	0	0	No	—	0	0	0
68	First batch	Marfan syndrome	No	—	0	0	0	No	—	0	0	0

69	First batch	McCune-albright syndrome	No	—	0	0	0	No	—	0	0	0
70	First batch	Medium chain acyl-CoA dehydrogenase deficiency	No	—	0	0	0	No	—	0	0	0
71	First batch	Methylmalonic academia	No	—	0	0	0	No	—	0	0	0
72	First batch	Mitochodrial encephalomyopathy	No	—	0	0	0	No	—	0	0	0
73	First batch	Mucopolysaccharidosis	Yes	Imported	2	0	2	Yes	Domestic	5	5	0
74	First batch	Multi-Focal motor neurothy	No	—	0	0	0	Yes	Domestic	1	1	0
75	First batch	Multiple acyl-CoA dehydrogenase deficiency	No	—	0	0	0	Yes	—	0	0	0
76	First batch	Multiple sclerosis	Yes	Domestic and imported	40	19	21	Yes	Domestic	8	8	0
77	First batch	Multiple system atrophy	No	—	0	0	0	No	—	0	0	0
78	First batch	Myotonic dystrophy	No	—	0	0	0	No	—	0	0	0
79	First batch	NAGS deficiency	No	—	0	0	0	No	—	0	0	0
80	First batch	Neonatal diabetes mellitus	No	—	0	0	0	No	—	0	0	0
81	First batch	Neuromyelitis optica	Yes	Imported	3	0	3	Yes	Domestic and imported	4	3	1
82	First batch	Niemann-Pick disease	Yes	Imported	1	0	1	Yes	Domestic	3	2	1

									and imported			
83	First batch	Non-Syndromic deafness	No	—	0	0	0	No	—	0	0	0
84	First batch	Noonan syndrome	Yes	Domestic and imported	38	36	2	Yes	Imported	1	0	1
85	First batch	Ornithine transcarbamylase deficiency	Yes	Domestic and imported	2	1	1	Yes	Domestic	1	1	0
86	First batch	Osteogenesis imperfecta（brittle bone disease）	No	—	0	0	0	No	—	0	0	0
87	First batch	Parkinson disease（young-onset , early-onset）	Yes	Domestic and imported	181	148	33	Yes	Domestic and imported	5	4	1
88	First batch	Paroxysmal nocturnal hemoglobinuia	Yes	Imported	3	0	3	Yes	Domestic and imported	9	8	1
89	First batch	Peutz-Jeghers syndrome	No	—	0	0	0	No	—	0	0	0
90	First batch	Phenylketouria	No	—	0	0	0	Yes	Domestic	2	2	0
91	First batch	POEMS syndrome	No	—	0	0	0	No	—	0	0	0
92	First batch	Porphyria	No	—	0	0	0	Yes	Domestic	3	3	0
93	First batch	Prader-Willi syndrome	Yes	Domestic and imported	38	36	2	Yes	Domestic	2	2	0
94	First batch	Primary combined immune deficiency	No	—	0	0	0	No	—	0	0	0

95	First batch	Primary hereditary dystonia	No	—	0	0	0	Yes	Domestic	3	3	0
96	First batch	Primary light chain amyloidosis	Yes	Imported	1	0	1	Yes	Domestic	1	1	0
97	First batch	Progressive familial intrahepatic cholestasis	Yes	Imported	4	0	4	Yes	Domestic and imported	3	2	1
98	First batch	Progressive muscular dystrophyies	Yes	Domestic	3	3	0	No	—	0	0	0
99	First batch	Propionic acidemia	No	—	0	0	0	No	—	0	0	0
100	First batch	Pulmonary alveolar proteinosis	No	—	0	0	0	No	—	0	0	0
101	First batch	Pulmonary cystic fibrosis	No	—	0	0	0	Yes	Domestic and imported	28	27	1
102	First batch	Retinitis pigmentosa	No	—	0	0	0	No	—	0	0	0
103	First batch	Retinoblastoma	No	Domestic	3	3	0	No	—	0	0	0
104	First batch	Severe congenital neutropenia	No	—	0	0	0	Yes	Domestic	5	5	0
105	First batch	Severe myoclonic epilepsy in infaricy （dravet syndrome）	Yes	Domestic	2	2	0	Yes	Imported	5	0	5
106	First batch	Sickle cell disease	No	—	0	0	0	Yes	Domestic and imported	10	7	3
107	First batch	Silver-Russell Syndrome	No	—	0	0	0	No	—	0	0	0
108	First batch	Sitosterolemia	Yes	Domestic	16	13	3	No	—	0	0	0

				and imported								
109	First batch	Spinal and bulbar muscular atrophy （kennedy disease）	No	—	0	0	0	No	—	0	0	0
110	First batch	Spinal muscular atrophy	Yes	Imported	3	0	3	Yes	Domestic	5	5	0
111	First batch	Spinocerebellar ataxia	No	—	0	0	0	No	—	0	0	0
112	First batch	Systemic sclerosis	Yes	Domestic and imported	6	4	2	Yes	Domestic and imported	2	1	1
113	First batch	Tetrahydrobiopterin deficiency	Yes	Domestic	1	1	0	Yes	Domestic	1	1	0
114	First batch	Tuberous sclerosis complex	Yes	Domestic and imported	11	7	4	Yes	Domestic and imported	5	1	4
115	First batch	Tyrosinemia	Yes	Imported	8	0	8	Yes	Imported	2	0	2
116	First batch	Very long chain acyl-CoA dehydrogenase deficiency	No	—	0	0	0	No	—	0	0	0
117	First batch	Williams syndrome	No	—	0	0	0	No	—	0	0	0
118	First batch	Wiskott-Aldrich syndrome	Yes	Domestic and imported	13	12	1	Yes	Imported	1	0	1
119	First batch	X-linked agammaglobulinemia	No	—	0	0	0	No	—	0	0	0
120	First batch	X-linked ldrenoleuko dystrophy	No	—	0	0	0	No	—	0	0	0

121	First batch	X-linked lymphoproliferative disease	No	—	0	0	0	No	—	0	0	0
122	Second batch	Achondroplasia	Yes	Domestic and imported	38	36	2	Yes	Domestic	2	2	0
123	Second batch	Acquired hemophilia	Yes	Domestic and imported	5	1	4	Yes	Domestic and imported	28	18	10
124	Second batch	Acromegaly	Yes	Domestic and imported	54	45	9	Yes	Domestic and imported	5	1	4
125	Second batch	Adult-onset still disease	Yes	Imported	1	0	1	Yes	Imported	1	0	1
126	Second batch	Alagille syndrome	Yes	Imported	1	0	1	Yes	Domestic and imported	3	2	1
127	Second batch	Alpha-1-antitrypsin deficiency	No	—	0	0	0	No	—	0	0	0
128	Second batch	ANCA-associated vasculitis	Yes	Imported	1	0	1	Yes	Domestic and imported	5	3	2
129	Second batch	Bardet-Biedl syndrome	No	—	0	0	0	Yes	Domestic	2	2	0
130	Second batch	Behçet's disease	No	—	0	0	0	No	—	0	0	0
131	Second batch	Blue rubber bleb nevus	No	—	0	0	0	No	—	0	0	0
132	Second batch	CDKL5-deficiency disorder	Yes	Imported	1	0	1	Yes	Domestic	1	1	0
133	Second batch	Choroideremia	No	—	0	0	0	No	—	0	0	0
134	Second batch	Chronic inflammatory	Yes	Imported	1	0	1	Yes	Domestic	5	1	4

		demyelinating polyneuropathy							and imported			
135	Second batch	Clear cell sarcoma of kidney	Yes	Domestic	3	3	0	No	—	0	0	0
136	Second batch	Cold agglutinin disease	Yes	Domestic and imported	12	10	2	No	—	0	0	0
137	Second batch	Congenital biliary atresia	No	—	0	0	0	No	—	0	0	0
138	Second batch	Congenital factor VII deficiency	Yes	Imported	3	0	3	Yes	Imported	2	0	2
139	Second batch	Cryopyrin associated periodic syndrome/ NLRP3-associated systemic autoinflammatory disease	Yes	Imported	1	0	1	Yes	Domestic	1	1	0
140	Second batch	Cutaneous neuroendocrine carcinoma(Merkel cell carcinoma)	No	—	0	0	0	Yes	Domestic and imported	3	2	1
141	Second batch	Cutaneous T-cell lymphomas	Yes	Imported	2	0	2	Yes	Domestic and imported	18	16	2
142	Second batch	Cystinosis	No	—	0	0	0	Yes	Domestic	6	6	0
143	Second batch	Dermatofibrosarcoma protuberans	Yes	Domestic and imported	17	16	1	Yes	Imported	1	0	1

144	Second batch	Eosinophilic gastroenteritis	No	—	0	0	0	No	—	0	0	0
145	Second batch	Epithelioid sarcoma	No	—	0	0	0	No	—	0	0	0
146	Second batch	Facioscapulohumeral muscular dystrophy	No	—	0	0	0	No	—	0	0	0
147	Second batch	Familial hemophagocytic lymphohistiocytosis	Yes	Imported	3	0	3	Yes	Imported	1	0	1
148	Second batch	Familial adenomatous polyposis	No	—	0	0	0	No	—	0	0	0
149	Second batch	Fibrodysplasia ossificans progressiva	No	—	0	0	0	Yes	Imported	1	0	1
150	Second batch	Fragile X syndrome	No	—	0	0	0	No	—	0	0	0
151	Second batch	Gangliosidosis	No	—	0	0	0	No	—	0	0	0
152	Second batch	Gastroenteropancreatic neuroendocrine neoplasm	Yes	Domestic and imported	53	47	6	Yes	Imported	2	0	2
153	Second batch	Gastrointestinal stromal tumor	Yes	Domestic and imported	54	38	16	Yes	Domestic and imported	5	2	3
154	Second batch	Generalized pustular psoriasis	Yes	Imported	2	0	2	Yes	Imported	2	0	2
155	Second batch	Genetic hypoparathyroidism	No	—	0	0	0	Yes	Domestic and imported	3	2	1
156	Second batch	Giant cell arteritis	Yes	Imported	1	0	1	Yes	Domestic	1	1	0
157	Second batch	Giant cell tumor of bone	Yes	Domestic and	8	7	1	Yes	Domestic	1	1	0

				imported								
158	Second batch	Glanzmann thrombasthenia	Yes	Imported	3	0	3	Yes	Imported	1	0	1
159	Second batch	Glioblastoma	Yes	Domestic and imported	35	30	5	Yes	Domestic	2	2	0
160	Second batch	Gorlin syndrome	No	—	0	0	0	No	—	0	0	0
161	Second batch	Hidradenitis suppurativa	No	—	0	0	0	Yes	Domestic	2	2	0
162	Second batch	Hutchinson-Gilford progeria syndrome	No	—	0	0	0	Yes	Domestic	1	1	0
163	Second batch	Inflammatory myofibroblastic tumor	No	—	0	0	0	Yes	Domestic	2	2	0
164	Second batch	Leber congenital amaurosis	No	—	0	0	0	No	—	0	0	0
165	Second batch	Lennox-Gastaut syndrome	Yes	Domestic and imported	32	20	12	Yes	Domestic and imported	8	2	6
166	Second batch	Limbal stem cell deficiency	No	—	0	0	0	No	—	0	0	0
167	Second batch	Malignant hyperthermia	Yes	Domestic	1	1	0	Yes	Domestic	1	1	0
168	Second batch	Malignant pleural mesothelioma	Yes	Domestic and imported	63	56	7	Yes	Domestic	1	1	0
169	Second batch	Melanoma	Yes	Domestic and imported	24	18	6	Yes	Domestic and imported	35	26	9
170	Second batch	Metachromatic	No	—	0	0	0	Yes	Imported	1	0	1

		leukodystrophy										
171	Second batch	Monogenic non-syndromic obesity	No	—	0	0	0	No	—	0	0	0
172	Second batch	Multiple endocrine neoplasia	Yes	Domestic	2	2	0	No	—	0	0	0
173	Second batch	Narcolepsy	Yes	Domestic and imported	4	2	2	Yes	Domestic and imported	9	3	6
174	Second batch	Neuroblastoma	Yes	Imported	2	0	2	Yes	Domestic	4	4	0
175	Second batch	Neurofibromatosis	Yes	Domestic and imported	4	2	2	Yes	Domestic and imported	2	1	1
176	Second batch	Neuronal ceroid lipofuscinosis	No	—	0	0	0	Yes	Domestic	2	2	0
177	Second batch	Neurotrophic keratitis	Yes	Imported	1	0	1	Yes	Imported	1	0	1
178	Second batch	Osteosarcoma	No	—	0	0	0	Yes	Domestic	2	2	0
179	Second batch	Pemphigus	No	—	0	0	0	Yes	Domestic	1	1	0
180	Second batch	Persistent pulmonary hypertension of the newborn	Yes	Imported	1	0	1	Yes	Domestic	2	2	0
181	Second batch	Pheochromocytoma	No	—	0	0	0	Yes	Domestic	2	2	0
182	Second batch	PIK3CA related overgrowth syndrome	No	—	0	0	0	Yes	Imported	2	0	2
183	Second batch	Polycythaemia vera	Yes	Domestic and imported	11	4	7	Yes	Domestic and imported	3	2	1
184	Second batch	Primary biliary cholangitis	No	—	0	0	0	Yes	Domestic and	2	1	1

									imported			
185	Second batch	Primary ciliary dyskinesia	Yes	Domestic and imported	40	38	2	Yes	Domestic and imported	14	8	6
186	Second batch	Primary IGF1 deficiency	No	—	0	0	0	No	—	0	0	0
187	Second batch	Primary immunodeficiency	Yes	Domestic	115	115	0	Yes	Imported	1	0	1
188	Second batch	Primary myelofibrosis	Yes	Domestic and imported	10	4	6	Yes	Domestic and imported	4	3	1
189	Second batch	Primary sclerosing cholangitis	Yes	Domestic and imported	13	12	1	No	—	0	0	0
190	Second batch	Progressive fibrosing interstitial lung disease	Yes	Domestic and imported	10	8	2	Yes	Imported	1	0	1
191	Second batch	Recurrent pericarditis	Yes	Imported	1	0	1	Yes	Domestic	1	1	0
192	Second batch	Retinopathy of prematurity	Yes	Domestic and imported	4	1	3	Yes	Domestic	1	1	0
193	Second batch	Rett syndrome	No	—	0	0	0	Yes	Domestic	2	2	0
194	Second batch	Short bowel syndrome	Yes	Imported	1	0	1	Yes	Domestic and imported	4	3	1
195	Second batch	Systemic juvenile idiopathic arthritis	Yes	Domestic and imported	9	6	3	Yes	Domestic	5	5	0

196	Second batch	Systemic mastocytosis	No	—	0	0	0	Yes	Domestic and imported	5	2	3
197	Second batch	Takayasu arteritis	No	—	0	0	0	No	—	0	0	0
198	Second batch	Tenosynovial giant cell tumor/Pigmented villonodular synovitis	Yes	Domestic	1	1	0	Yes	Imported	1	0	1
199	Second batch	Thalassemia major	Yes	Domestic and imported	11	3	8	Yes	Domestic and imported	5	3	2
200	Second batch	Thrombotic thrombocytopenic purpura	Yes	Imported	1	0	1	Yes	Domestic and imported	3	1	2
201	Second batch	Transthyretin amyloidosis	Yes	Domestic and imported	5	3	2	Yes	Domestic	7	7	0
202	Second batch	Tumor necrosis factor receptor associated periodic syndrome	No	—	0	0	0	No	—	0	0	0
203	Second batch	Tumor-induced osteomalacia	Yes	Imported	3	0	3	Yes	Imported	1	0	1
204	Second batch	Von Hippel-Lindau syndrome	Yes	Imported	1	0	1	Yes	Domestic	1	1	0
205	Second batch	Von Willebrand disease type3	Yes	Domestic and imported	15	12	3	Yes	Domestic and imported	3	1	2
206	Second batch	Waldenström macroglobulinemia/	Yes	Domestic and	8	6	2	Yes	Domestic and	10	8	2

		Lymphoplasmacytic lymphoma		imported					imported			
207	Second batch	West syndrome/Infantile spasms syndrome	Yes	Domestic and imported	4	2	2	Yes	Domestic and imported	3	2	1

Note: The data in this table is as of December 31, 2025. Data sourced from the Yozhi data website.

Table S4. Analysis of the availability of rare disease drugs in China and the United States before the catalog release

No.	Batches	Disease name	China					United States				
			**Drugs availabl e**	**Source of drugs**	**Number of drug approvals (all)**	**Number of domestic drug approvals**	**Number of imported drug approvals**	**Drugs available**	**Source of Drugs**	**Number of drug approvals (all)**	**Number of domestic drug approvals**	**Number of imported drug approvals**
1	First batch	21-Hydroxyulase deficiency	No	—	0	0	0	No	—	0	0	0
2	First batch	Albinism	No	—	0	0	0	No	—	0	0	0
3	First batch	Alport syndrome	No	—	0	0	0	No	—	0	0	0
4	First batch	Amyotrophic lateral sclerosis	Yes	Domestic and imported	21	19	2	Yes	Domestic and imported	2	1	1
5	First batch	Angelman syndrome	No	—	0	0	0	No	—	0	0	0
6	First batch	Arginase deficiency	No	—	0	0	0	No	—	0	0	0
7	First batch	Asphyxiating thoracic dystrophy（Jeune syndrome）	No	—	0	0	0	No	—	0	0	0
8	First batch	Atypical hemolytic	No	—	0	0	0	Yes	Domestic	1	1	0

		uremic syndrome										
9	First batch	Autoimmune encephalitis	No	—	0	0	0	No	—	0	0	0
10	First batch	Autoimmune hypophysitis	No	—	0	0	0	No	—	0	0	0
11	First batch	Autoimmune insulin receptopathy（type B insulin resistance）	No	—	0	0	0	No	—	0	0	0
12	First batch	Beta-ketothiolase deficiency	No	—	0	0	0	No	—	0	0	0
13	First batch	Biotinidase deficiency	No	—	0	0	0	No	—	0	0	0
14	First batch	Cardic ion channelopathies	No	—	0	0	0	No	—	0	0	0
15	First batch	Carnitine deficiency	Yes	Domestic and imported	42	41	1	Yes	Domestic	3	3	0
16	First batch	Castleman disease	No	—	0	0	0	Yes	Domestic	1	1	0
17	First batch	Charcot-marie-tooth disease	No	—	0	0	0	No	—	0	0	0
18	First batch	Citrullinemia	No	—	0	0	0	No	—	0	0	0
19	First batch	Congenital adrenal hypoplasia	No	—	0	0	0	No	—	0	0	0
20	First batch	Congenital hyperinsulinemic hypoglycemia	No	—	0	0	0	No	—	0	0	0
21	First batch	Congenital myasthenic syndrome	No	—	0	0	0	No	—	0	0	0
22	First batch	Congenital myotonia	No	—	0	0	0	No	—	0	0	0

		syndrome（Non-dystrophic myotonia, NDM）										
23	First batch	Congenital scoliosis	No	—	0	0	0	No	—	0	0	0
24	First batch	Coronary artery ectasia	No	—	0	0	0	No	—	0	0	0
25	First batch	Diamond-blackfan anemia	No	—	0	0	0	No	—	0	0	0
26	First batch	Erdheim-chester disease	No	—	0	0	0	Yes	Domestic	1	1	0
27	First batch	Fabry disease	No	—	0	0	0	Yes	Domestic	1	1	0
28	First batch	Familial mediterranean fever	No	—	0	0	0	Yes	Domestic	2	2	0
29	First batch	Fanconi anemia	No	—	0	0	0	No	—	0	0	0
30	First batch	Galactosemia	No	—	0	0	0	No	—	0	0	0
31	First batch	Gaucher’s disease	Yes	Imported	1	0	1	Yes	Domestic	6	6	0
32	First batch	General myathenic gravis	No	—	0	0	0	Yes	Domestic and imported	1	1	0
33	First batch	Gitelman syndrome	No	—	0	0	0	No	—	0	0	0
34	First batch	Glutaric acidemia type I	No	—	0	0	0	No	—	0	0	0
35	First batch	Glycogen storage disease（type I、II）	Yes	Imported	1	0	1	Yes	Domestic	3	3	0
36	First batch	Hemophilia	Yes	Domestic and imported	59	40	19	Yes	Domestic	15	15	0
37	First batch	Hepatolenticular degeneration（wilson disease）	Yes	Domestic and imported	8	8	0	Yes	Domestic and imported	2	2	0
38	First batch	Hereditary angioedema	Yes	Domestic	9	8	1	Yes	Domestic	7	6	1

		（HAE）		and imported					and imported			
39	First batch	Hereditary epidermolysis bullosa	No	—	0	0	0	No	—	0	0	0
40	First batch	Hereditary fructose intolerance	No	—	0	0	0	No	—	0	0	0
41	First batch	Hereditary hypomagnesemia	Yes	Domestic	6	6	0	No	—	0	0	0
42	First batch	Hereditary multi-infarct dementia（Cerebral autosomal dominant arteriopathy with subcortical infarcts and leukoencephalopathy, CADASIL）	No	—	0	0	0	No	—	0	0	0
43	First batch	Hereditary spastic paraplegia	No	—	0	0	0	No	—	0	0	0
44	First batch	Holocarboxylase synthetase deficiency	No	—	0	0	0	No	—	0	0	0
45	First batch	Homocysteinemia	No	—	0	0	0	No	—	0	0	0
46	First batch	Homozygous hypercholesterolemia	Yes	Domestic and imported	174	150	24	Yes	Domestic and imported	4	3	1
47	First batch	Huntington disease	No	—	0	0	0	Yes	Domestic and imported	2	1	1
48	First batch	Hyperornithinaemia-hyperammonaemia-hhomo	No	—	0	0	0	No	—	0	0	0

		citrullinuria syndrome										
49	First batch	Hyperphenylalaninemia	No	—	0	0	0	Yes	Domestic	1	1	0
50	First batch	Hypophosphatasia	No	—	0	0	0	Yes	Domestic	1	1	0
51	First batch	Hypophosphatemic rickets	Yes	Domestic and imported	5	3	2	Yes	Imported	1	0	1
52	First batch	Idiopathic Cardiomyopathy	No	—	0	0	0	Yes	Domestic	1	1	0
53	First batch	Idiopathic hypogonadotropic hypogonadism	Yes	Domestic and imported	52	51	1	Yes	Imported	2	0	2
54	First batch	Idiopathic pulmonary arterial hypertension	Yes	Imported	9	0	9	Yes	Domestic and imported	11	6	5
55	First batch	Idiopathic pulmonary fibrosis	Yes	Domestic and imported	4	2	2	Yes	Domestic and imported	2	1	1
56	First batch	IgG4 related disease	No	—	0	0	0	No	—	0	0	0
57	First batch	Inborn errors of bile acid synthesis	No	—	0	0	0	Yes	Domestic	1	1	0
58	First batch	Isovaleric acidemia	No	—	0	0	0	No	—	0	0	0
59	First batch	Kallmann syndrome	No	—	0	0	0	No	—	0	0	0
60	First batch	Langerhans cell histiocytosis	No	—	0	0	0	No	—	0	0	0
61	First batch	Laron syndrome	No	—	0	0	0	No	—	0	0	0
62	First batch	Leber hereditary optic neuropathy	No	—	0	0	0	No	—	0	0	0
63	First batch	Long chain	No	—	0	0	0	No	—	0	0	0

		3-hydroxyacyl-CoA dehydrogenase deficiency										
64	First batch	Lymphangioleiomyomatosis (LAM)	No	—	0	0	0	Yes	Domestic and imported	4	1	3
65	First batch	Lysine urinary protein intolerance	No	—	0	0	0	No	—	0	0	0
66	First batch	Lysosomal acid lipase deficiency	No	—	0	0	0	Yes	Domestic	1	1	0
67	First batch	Maple syrup urine disease	No	—	0	0	0	No	—	0	0	0
68	First batch	Marfan syndrome	No	—	0	0	0	No	—	0	0	0
69	First batch	McCune-albright syndrome	No	—	0	0	0	No	—	0	0	0
70	First batch	Medium chain acyl-CoA dehydrogenase deficiency	No	—	0	0	0	No	—	0	0	0
71	First batch	Methylmalonic academia	No	—	0	0	0	No	—	0	0	0
72	First batch	Mitochodrial encephalomyopathy	No	—	0	0	0	No	—	0	0	0
73	First batch	Mucopolysaccharidosis	No	—	0	0	0	Yes	Domestic	5	5	0
74	First batch	Multi-Focal motor neurothy	No	—	0	0	0	Yes	Domestic	1	1	0
75	First batch	Multiple acyl-CoA dehydrogenase	No	—	0	0	0	Yes	—	0	0	0

		deficiency										
76	First batch	Multiple sclerosis	Yes	Domestic and imported	9	6	3	Yes	Domestic	7	7	0
77	First batch	Multiple system atrophy	No	—	0	0	0	No	—	0	0	0
78	First batch	Myotonic dystrophy	No	—	0	0	0	No	—	0	0	0
79	First batch	NAGS deficiency	No	—	0	0	0	No	—	0	0	0
80	First batch	Neonatal diabetes mellitus	No	—	0	0	0	No	—	0	0	0
81	First batch	Neuromyelitis optica	No	—	0	0	0	Yes	Domestic and imported	0	0	0
82	First batch	Niemann-Pick disease	Yes	Imported	1	0	1	Yes	Domestic and imported	0	0	0
83	First batch	Non-Syndromic deafness	No	—	0	0	0	No	—	0	0	0
84	First batch	Noonan syndrome	Yes	Domestic and imported	30	28	2	Yes	Imported	1	0	1
85	First batch	Ornithine transcarbamylase deficiency	Yes	Domestic and imported	0	0	0	Yes	Domestic	1	1	0
86	First batch	Osteogenesis imperfecta（brittle bone disease）	No	—	0	0	0	No	—	0	0	0
87	First batch	Parkinson disease（young-onset ,	Yes	Domestic and	87	73	14	Yes	Domestic and	3	2	1

		early-onset）		imported					imported			
88	First batch	Paroxysmal nocturnal hemoglobinuia	No	—	0	0	0	Yes	Domestic	1	1	0
89	First batch	Peutz-Jeghers syndrome	No	—	0	0	0	No	—	0	0	0
90	First batch	Phenylketouria	No	—	0	0	0	Yes	Domestic	1	1	0
91	First batch	POEMS syndrome	No	—	0	0	0	No	—	0	0	0
92	First batch	Porphyria	No	—	0	0	0	Yes	Domestic	1	1	0
93	First batch	Prader-Willi syndrome	Yes	Domestic and imported	30	28	2	Yes	Domestic	1	1	0
94	First batch	Primary combined immune deficiency	No	—	0	0	0	No	—	0	0	0
95	First batch	Primary hereditary dystonia	No	—	0	0	0	Yes	Domestic	2	2	0
96	First batch	Primary light chain amyloidosis	No	—	0	0	0	No	—	0	0	0
97	First batch	Progressive familial intrahepatic cholestasis	No	—	0	0	0	No	—	0	0	0
98	First batch	Progressive muscular dystrophyies	Yes	Domestic	3	3	0	No	—	0	0	0
99	First batch	Propionic acidemia	No	—	0	0	0	No	—	0	0	0
100	First batch	Pulmonary alveolar proteinosis	No	—	0	0	0	No	—	0	0	0
101	First batch	Pulmonary cystic fibrosis	No	—	0	0	0	Yes	Domestic	12	12	0
102	First batch	Retinitis pigmentosa	No	—	0	0	0	No	—	0	0	0
103	First batch	Retinoblastoma	No	—	0	0	0	No	—	0	0	0
104	First batch	Severe congenital	No	—	0	0	0	Yes	Domestic	5	5	0

		neutropenia										
105	First batch	Severe myoclonic epilepsy in infaricy （dravet syndrome）	No	—	0	0	0	No	—	0	0	0
106	First batch	Sickle cell disease	No	—	0	0	0	Yes	Domestic	3	3	0
107	First batch	Silver-Russell Syndrome	No	—	0	0	0	No	—	0	0	0
108	First batch	Sitosterolemia	Yes	Imported	1	0	1	No	—	0	0	0
109	First batch	Spinal and bulbar muscular atrophy （kennedy disease）	No	—	0	0	0	No	—	0	0	0
110	First batch	Spinal muscular atrophy	No	—	0	0	0	Yes	Domestic	1	1	0
111	First batch	Spinocerebellar ataxia	No	—	0	0	0	No	—	0	0	0
112	First batch	Systemic sclerosis	No	—	0	0	0	No	—	0	0	0
113	First batch	Tetrahydrobiopterin deficiency	No	—	0	0	0	Yes	Domestic	1	1	0
114	First batch	Tuberous sclerosis complex	Yes	Imported	3	0	3	Yes	Imported	3	0	3
115	First batch	Tyrosinemia	No	—	0	0	0	Yes	Imported	2	0	2
116	First batch	Very long chain acyl-CoA dehydrogenase deficiency	No	—	0	0	0	No	—	0	0	0
117	First batch	Williams syndrome	No	—	0	0	0	No	—	0	0	0
118	First batch	Wiskott-Aldrich syndrome	Yes	Imported	1	0	1	Yes	Imported	1	0	1
119	First batch	X-linked agammaglobulinemia	No	—	0	0	0	No	—	0	0	0

120	First batch	X-linked ldrenoleuko dystrophy	No	—	0	0	0	No	—	0	0	0
121	First batch	X-linked lymphoproliferative disease	No	—	0	0	0	No	—	0	0	0
122	Second batch	Achondroplasia	Yes	Domestic and imported	34	32	2	Yes	Domestic	1	1	0
123	Second batch	Acquired hemophilia	Yes	Domestic and imported	3	0	3	Yes	Domestic and imported	22	13	9
124	Second batch	Acromegaly	Yes	Domestic and imported	49	40	9	Yes	Domestic and imported	5	1	4
125	Second batch	Adult-onset still disease	No	—	0	0	0	Yes	Imported	1	0	1
126	Second batch	Alagille syndrome	Yes	Imported	1	0	1	Yes	Domestic and imported	3	2	1
127	Second batch	Alpha-1-antitrypsin deficiency	No	—	0	0	0	No	—	0	0	0
128	Second batch	ANCA-associated vasculitis	No	—	0	0	0	Yes	Domestic and imported	5	3	2
129	Second batch	Bardet-Biedl syndrome	No	—	0	0	0	Yes	Domestic	1	1	0
130	Second batch	Behçet's disease	No	—	0	0	0	No	—	0	0	0
131	Second batch	Blue rubber bleb nevus	No	—	0	0	0	No	—	0	0	0
132	Second batch	CDKL5-deficiency disorder	No	—	0	0	0	Yes	Domestic	1	1	0

133	Second batch	Choroideremia	No	—	0	0	0	No	—	0	0	0
134	Second batch	Chronic inflammatory demyelinating polyneuropathy	No	—	0	0	0	Yes	Imported	2	0	2
135	Second batch	Clear cell sarcoma of kidney	Yes	Domestic	3	3	0	No	—	0	0	0
136	Second batch	Cold agglutinin disease	Yes	Domestic and imported	10	8	2	No	—	0	0	0
137	Second batch	Congenital biliary atresia	No	—	0	0	0	No	—	0	0	0
138	Second batch	Congenital factor VII deficiency	Yes	Imported	3	0	3	Yes	Imported	2	0	2
139	Second batch	Cryopyrin associated periodic syndrome/ NLRP3-associated systemic autoinflammatory disease	No	—	0	0	0	Yes	Domestic	1	1	0
140	Second batch	Cutaneous neuroendocrine carcinoma(Merkel cell carcinoma)	No	—	0	0	0	Yes	Domestic and imported	3	2	1
141	Second batch	Cutaneous T-cell lymphomas	Yes	Imported	2	0	2	Yes	Domestic and imported	18	16	2
142	Second batch	Cystinosis	No	—	0	0	0	Yes	Domestic	6	6	0
143	Second batch	Dermatofibrosarcoma	Yes	Domestic	15	14	1	Yes	Imported	1	0	1

		protuberans		and imported								
144	Second batch	Eosinophilic gastroenteritis	No	—	0	0	0	No	—	0	0	0
145	Second batch	Epithelioid sarcoma	No	—	0	0	0	No	—	0	0	0
146	Second batch	Facioscapulohumeral muscular dystrophy	No	—	0	0	0	No	—	0	0	0
147	Second batch	Familial hemophagocytic lymphohistiocytosis	Yes	Imported	3	0	3	Yes	Imported	1	0	1
148	Second batch	Familial adenomatous polyposis	No	—	0	0	0	No	—	0	0	0
149	Second batch	Fibrodysplasia ossificans progressiva	No	—	0	0	0	Yes	Imported	1	0	1
150	Second batch	Fragile X syndrome	No	—	0	0	0	No	—	0	0	0
151	Second batch	Gangliosidosis	No	—	0	0	0	No	—	0	0	0
152	Second batch	Gastroenteropancreatic neuroendocrine neoplasm	Yes	Domestic and imported	44	38	6	Yes	Imported	1	0	1
153	Second batch	Gastrointestinal stromal tumor	Yes	Domestic and imported	39	25	14	Yes	Domestic and imported	5	2	3
154	Second batch	Generalized pustular psoriasis	Yes	Imported	1	0	1	Yes	Imported	1	0	1
155	Second batch	Genetic hypoparathyroidism	No	—	0	0	0	Yes	Domestic and imported	2	1	1
156	Second batch	Giant cell arteritis	No	—	0	0	0	No	—	0	0	0

157	Second batch	Giant cell tumor of bone	Yes	Domestic and imported	4	3	1	Yes	Domestic	1	1	0
158	Second batch	Glanzmann thrombasthenia	Yes	Imported	3	0	3	Yes	Imported	1	0	1
159	Second batch	Glioblastoma	Yes	Domestic and imported	23	18	5	Yes	Domestic	2	2	0
160	Second batch	Gorlin syndrome	No	—	0	0	0	No	—	0	0	0
161	Second batch	Hidradenitis suppurativa	No	—	0	0	0	Yes	Domestic	2	2	0
162	Second batch	Hutchinson-Gilford progeria syndrome	No	—	0	0	0	Yes	Domestic	1	1	0
163	Second batch	Inflammatory myofibroblastic tumor	No	—	0	0	0	Yes	Domestic	2	2	0
164	Second batch	Leber congenital amaurosis	No	—	0	0	0	No	—	0	0	0
165	Second batch	Lennox-Gastaut syndrome	Yes	Domestic and imported	19	9	10	Yes	Domestic and imported	8	2	6
166	Second batch	Limbal stem cell deficiency	No	—	0	0	0	No	—	0	0	0
167	Second batch	Malignant hyperthermia	Yes	Domestic	1	1	0	Yes	Domestic	1	1	0
168	Second batch	Malignant pleural mesothelioma	Yes	Domestic and imported	54	48	6	Yes	Domestic	1	1	0
169	Second batch	Melanoma	Yes	Domestic and	22	16	6	Yes	Domestic and	33	24	9

				imported					imported			
170	Second batch	Metachromatic leukodystrophy	No	—	0	0	0	No	—	0	0	0
171	Second batch	Monogenic non-syndromic obesity	No	—	0	0	0	No	—	0	0	0
172	Second batch	Multiple endocrine neoplasia	Yes	Domestic	2	2	0	No	—	0	0	0
173	Second batch	Narcolepsy	Yes	Domestic and imported	4	2	2	Yes	Domestic and imported	9	3	6
174	Second batch	Neuroblastoma	Yes	Imported	2	0	2	Yes	Domestic	3	3	0
175	Second batch	Neurofibromatosis	Yes	Imported	2	0	2	Yes	Domestic and imported	1	0	1
176	Second batch	Neuronal ceroid lipofuscinosis	No	—	0	0	0	Yes	Domestic	1	1	0
177	Second batch	Neurotrophic keratitis	Yes	Imported	1	0	1	Yes	Imported	1	0	1
178	Second batch	Osteosarcoma	No	—	0	0	0	Yes	Domestic	2	2	0
179	Second batch	Pemphigus	No	—	0	0	0	Yes	Domestic	1	1	0
180	Second batch	Persistent pulmonary hypertension of the newborn	Yes	Imported	1	0	1	Yes	Domestic	2	2	0
181	Second batch	Pheochromocytoma	No	—	0	0	0	Yes	Domestic	2	2	0
182	Second batch	PIK3CA related overgrowth syndrome	No	—	0	0	0	Yes	Imported	1	0	1
183	Second batch	Polycythaemia vera	Yes	Imported	6	0	6	Yes	Domestic and imported	3	2	1

184	Second batch	Primary biliary cholangitis	No	—	0	0	0	Yes	Domestic and imported	0	0	0
185	Second batch	Primary ciliary dyskinesia	Yes	Domestic and imported	35	33	2	Yes	Domestic and imported	14	8	6
186	Second batch	Primary IGF1 deficiency	No	—	0	0	0	No	—	0	0	0
187	Second batch	Primary immunodeficiency	Yes	Domestic	115	115	0	Yes	Imported	1	0	1
188	Second batch	Primary myelofibrosis	Yes	Imported	6	0	6	Yes	Domestic and imported	4	3	1
189	Second batch	Primary sclerosing cholangitis	Yes	Domestic and imported	4	3	1	No	—	0	0	0
190	Second batch	Progressive fibrosing interstitial lung disease	Yes	Domestic and imported	8	6	2	Yes	Imported	1	0	1
191	Second batch	Recurrent pericarditis	No	—	0	0	0	Yes	Domestic	1	1	0
192	Second batch	Retinopathy of prematurity	Yes	Imported	3	0	3	No	—	0	0	0
193	Second batch	Rett syndrome	No	—	0	0	0	Yes	Domestic	1	1	0
194	Second batch	Short bowel syndrome	No	—	0	0	0	Yes	Domestic and imported	4	3	1
195	Second batch	Systemic juvenile idiopathic arthritis	Yes	Domestic and	6	3	3	Yes	Domestic	4	4	0

				imported								
196	Second batch	Systemic mastocytosis	No	—	0	0	0	Yes	Domestic and imported	5	2	3
197	Second batch	Takayasu arteritis	No	—	0	0	0	No	—	0	0	0
198	Second batch	Tenosynovial giant cell tumor/Pigmented villonodular synovitis	No	—	0	0	0	Yes	Imported	1	0	1
199	Second batch	Thalassemia major	Yes	Domestic and imported	9	1	8	Yes	Domestic and imported	4	2	2
200	Second batch	Thrombotic thrombocytopenic purpura	No	—	0	0	0	Yes	Imported	1	0	1
201	Second batch	Transthyretin amyloidosis	Yes	Imported	2	0	2	Yes	Domestic	4	4	0
202	Second batch	Tumor necrosis factor receptor associated periodic syndrome	No	—	0	0	0	No	—	0	0	0
203	Second batch	Tumor-induced osteomalacia	Yes	Imported	3	0	3	Yes	Imported	1	0	1
204	Second batch	Von Hippel-Lindau syndrome	No	—	0	0	0	Yes	Domestic	1	1	0
205	Second batch	Von Willebrand disease type3	Yes	Domestic and imported	10	9	1	Yes	Domestic and imported	3	1	2
206	Second batch	Waldenström macroglobulinemia/	Yes	Domestic and	3	2	1	Yes	Domestic and	8	6	2

		Lymphoplasmacytic lymphoma		imported					imported			
207	Second batch	West syndrome/Infantile spasms syndrome	Yes	Domestic and imported	3	1	2	Yes	Domestic and imported	2	1	1

Note: The first batch of the Rare Disease Catalog was released on May 11, 2018, and the second batch was released on September 18, 2018. The data in this table are as of the release dates of these two batches of the Rare Disease Catalog. Data sourced from the Yozhi data website.

Table S5. Analysis of IND and NDA types for drugs approved by NMPA from 2015 to 2025

Drug application types	Drug Type			P-value
	ALL	Domestic drugs No. (%)	Imported drugs No. (%)	
**Drug IND**				
Class I new drug	1041/2007 (51.87)	776/1331 (58.30)	265/676 (39.20)	<0.001
Others	966/2007 (48.13)	555/1331 (41.70)	411/676 (60.80)	
**Drug NDA**				
Class I new drug	27/1134 (2.38)	16/807 (1.98)	11/327 (3.36)	0.167
Others	1107/1134 (97.62)	791/807 (98.02)	316/327 (96.63)	

Note: Class I new drugs in this study refer to innovative drugs, including innovative chemical drugs and innovative therapeutic biological products. IND, Investigational New Drug; NDA, New Drug Application. The Fisher's precision probability test was used to analyze the distribution of each categorical variable.

Table S6. Information on drugs corresponding to diseases listed in the Rare Disease Catalog that were not covered by medical insurance.

NO.	Batches	Disease name	Drug name	Drug specifications	Manufacturer	Approval Number	Drug source	Unit price (RMB)
1	First batch	Arginase Deficiency	Glycerol Phenylbutyrate Oral Liquid	25ml	Unimedic AB	HJ20230071	Imported	3700
2	First batch	Citrullinemia	Glycerol Phenylbutyrate Oral Liquid	25ml	Unimedic AB	HJ20230071	Imported	3700
3	First batch	Familial Mediterranean Fever	Anakinra injection	100 mg (0.67 mL)/vial	Hospira Zagreb d.o.o.	SJ20230016	Imported	265
4	First batch	Glycogen Storage Disease（Type I、II）	Avalglucosidase Alfa for Injection	100 mg/bottle	Genzyme Ireland Limited	SJ20230015	Imported	/
			Alglucosidase Alfa for Injection	50 mg/bottle	Genzyme Ireland Limited	SJ20150049	Imported	4980
5	First batch	Hyperornithinaemia-Hyperammonaemia-Hhomocitrullinuria Syndrome	Glycerol Phenylbutyrate Oral Liquid	25ml	Unimedic AB	HJ20230071	Imported	3700
6	First batch	Hyperphenylalaninemia	Sapropterin Dihydrochloride Tablets	100mg	Shandong New Time Pharmaceutical Co. Ltd	H20233796	Domestic	240.63
7	First batch	IgG4 related Disease	Inebilizumab Injection	100mg(10mL)/bottle	AstraZeneca Nijmegen B.V.	SJ20220007	Imported	21137.5
8	First batch	Isovaleric Acidemia	Carglumic Acid Dispersible Tablets	200mg	Grand Pharma (China) Co., Ltd	H20234128	Domestic	298
			Carglumic Acid Dispersible Tablets	200mg	Laboratoires BTT	HJ20230085	Imported	420
9	First batch	Mucopolysaccharidosis	Laronidase concentrated solution for infusion	500U (5 ml)/bottle	Vetter Pharma-Fertigung GmbH & Co. KG	SJ20200010	Imported	8100
			Idursulfase beta Injection	6 mg (3 ml)/bottle	GC Biopharma Corp.	SJ20200022	Imported	13888.89
10	First batch	Ornithine	Sodium Phenylbutyrate	150 g/bottle	Zhaoke Pharmaceutical	H20213365	Domestic	12620

		Transcarbamylase Deficiency	Granules		(Guangzhou) Ltd			
			Glycerol Phenylbutyrate Oral Liquid	25ml	Unimedic AB	HJ20230071	Imported	3700
11	First batch	Progressive Familial Intrahepatic Cholestasis	Odevixibat Capsules	1200μg	Patheon France	HJ20240147	Imported	/
			Odevixibat Capsules	600μg	Patheon France	HJ20240146	Imported	/
			Odevixibat Capsules	400μg	Patheon France	HJ20240148	Imported	1370
			Odevixibat Capsules	200μg	Patheon France	HJ20240145	Imported	805.88
12	First batch	Progressive Muscular Dystrophyies	Polysacharidum of G. Lucidum Karst Injection	2ml:4.5mg	Shanxi Guorun Pharmaceutical Co. Ltd.	H20003510	Domestic	168
			Polysacharidum of G.Lucidum Karst for Injection	4.5mg	Heilongjiang Jiangshi Pharmaceutical Co. Ltd.	H20051702	Domestic	2980
13	First batch	Tetrahydrobiopterin Deficiency	Sapropterin Dihydrochloride Tablets	100mg	Shandong New Time Pharmaceutical Co. Ltd	H20233796	Domestic	240.63
14	Second batch	Adult-onset Still disease	Anakinra injection	100 mg (0.67 ml)/vial	Hospira Zagreb d.o.o.	SJ20230016	Imported	265
15	Second batch	Alagille syndrome	Maralixibat Chloride Oral Solution	30ml：285mg	Halo Pharmaceutical Canada Inc.	HJ20230061	Imported	93750
16	Second batch	ANCA-associated vasculitis	Avacopan Capsules	10mg	Patheon Pharmaceuticals Inc.	HJ20240125	Imported	/
17	Second batch	CDKL5-deficiency disorder	Ganaxolone Oral Suspension	110ml：5.5g	Altasciences CDMO Philadelphia, LLC	SJ20240029	Imported	3550
18	Second batch	Chronic inflammatory demyelinating polyneuropathy	Efgartigimod Alfa Injection (Subcutaneous Injection)	1000mg(5.6ml)/ bottle	Patheon Italia S.p.A.	SJ20240029	Imported	9433
19	Second batch	Cryopyrin associated periodic syndrome/ NLRP3-associated systemic	Rilonacept for injection	220 mg/bottle	Jubilant HollisterStier LLC	SJ20240045	Imported	/

		autoinflammatory disease						
20	Second batch	Familial hemophagocytic lymphohistiocytosis	Emapalumab Injection	10 mg (2 ml)/ bottle	Patheon Italia S.p.A.	SJ20220008	Imported	4888
			Emapalumab Injection	50 mg (10 ml)/ bottle	Patheon Italia S.p.A.	SJ20220009	Imported	16757.8
21	Second batch	Giant cell arteritis	Upadacitinib Sustained-release Tablets	15mg	AbbVie Ireland NL B.V.	HJ20220010	Imported	65.48
22	Second batch	Malignant hyperthermia	Dantrolene Sodium for Injection	20mg	Livzon Pharmaceutical Group Inc	H20203530	Domestic	600
23	Second batch	Neuroblastoma	Naxitamab Injection	40 mg (10 ml)/bottle	Patheon Manufacturing Services LLC	SJ20220019	Imported	39500
			Dinutuximab beta Injection	20 mg (4.5 ml)/bottle	Patheon Italia S.P.A.	SJ20210023	Imported	59598
24	Second batch	Neurotrophic keratitis	Cenegermin eye drops	1ml: 20μg	Patheon Italia S.p.A.	SJ20200020	Imported	5232.14
25	Second batch	Persistent pulmonary hypertension of the newborn	Nitric Oxide for Inhalation	800ppm	Mallinckrodt Manufacturing LLC	HJ20220015	Imported	5693
26	Second batch	Recurrent pericarditis	Rilonacept for injection	220 mg/bottle	Jubilant HollisterStier LLC	SJ20240045	Imported	/
27	Second batch	Retinopathy of prematurity	Ranibizumab Injections	10mg/ml（0.20ml）	Qilu Pharmaceutical Co., Ltd	S20240034	Domestic	2570
			Ranibizumab Injections	10mg/ml（0.165ml）	Vetter Pharma-Fertigung GmbH & Co. KG；S.A. Alcon-Couvreur N.V.	SJ20181010	Imported	3673.5
			Ranibizumab Injections	10mg/ml（0.20ml）	Novartis Pharma Stein AG	SJ20170003	Imported	3673.5
28	Second batch	Short bowel syndrome	Teduglutide for Injection	5mg/bottle	Patheon Italia S.p.A.	SJ20240008	Imported	4637
29	Second batch	Tenosynovial giant cell tumor/Pigmented villonodular synovitis	Pimicotinib Hydrochloride Capsules	25 mg	Asymchem Life Science Tianjin Co., Ltd	H20250072	Domestic	/

30	Second batch	Thrombotic thrombocytopenic purpura	Caplacizumab for Injection	10mg/bottle	Patheon Italia S.p.A.	SJ20250023	Imported	/
31	Second batch	Tumor-induced osteomalacia	Burosumab Injection	10 mg (1 ml)/vial	Kyowa Kirin Co.,Ltd.,Takasaki Plant	SJ20210001	Imported	25888.92
			Burosumab Injection	20 mg (1 ml)/vial	Kyowa Kirin Co.,Ltd.,Takasaki Plant	SJ20210002	Imported	44011.17
			Burosumab Injection	30 mg (1 ml)/vial	Kyowa Kirin Co.,Ltd.,Takasaki Plant	SJ20210003	Imported	60029.8
32	Second batch	Von Hippel-Lindau syndrome	Belzutifan Tablets	40mg	MSD INTERNATIONAL GMBH	HJ20240135	Imported	884.44

Note: The drug price represents the winning bid price for this drug in Sichuan Province for 2025, retrieved via the YaoZhi Data website (https://www.yaozh.com). "/" indicates that price information for this drug was unavailable at the time of data query.
